# *Malva parviflora* extract ameliorates the deleterious effects of a high fat diet on the cognitive deficit in a mouse model of Alzheimer’s disease by restoring microglial function via a PPAR-γ-dependent mechanism

**DOI:** 10.1186/s12974-019-1515-3

**Published:** 2019-07-10

**Authors:** Elisa Medrano-Jiménez, Itzia Jiménez-Ferrer Carrillo, Martha Pedraza-Escalona, Cristina E. Ramírez-Serrano, Lourdes Álvarez-Arellano, Javier Cortés-Mendoza, Maribel Herrera-Ruiz, Enrique Jiménez-Ferrer, Alejandro Zamilpa, Jaime Tortoriello, Gustavo Pedraza-Alva, Leonor Pérez-Martínez

**Affiliations:** 1 Laboratorio de Neuroinmunobiología, Departamento de Medicina Molecular y Bioprocesos, Instituto de Biotecnología, Universidad Nacional Autónoma de México (UNAM), A.P. 510-3, CP, 62210 Cuernavaca, Morelos México; 2 Centro de Investigación Biomédica del Sur, Instituto Mexicano del Seguro Social (IMSS), Argentina No. 1, CP 62790 Xochitepec, Morelos México; 3 Present address: CONACYT-Hospital Infantil de México Federico Gómez, CP 06720 Ciudad de México, México

**Keywords:** Alzheimer’s Disease, obesity, inflammation, *Malva parviflora*, microglia, PPAR-γ/CD36

## Abstract

**Background:**

Alzheimer’s disease (AD) is a neuropathology strongly associated with the activation of inflammatory pathways. Accordingly, inflammation resulting from obesity exacerbates learning and memory deficits in humans and in animal models of AD. Consequently, the long-term use of non-steroidal anti-inflammatory agents diminishes the risk for developing AD, but the side effects produced by these drugs limit their prophylactic use. Thus, plants natural products have become an excellent option for modern therapeutics. *Malva parviflora* is a plant well known for its anti-inflammatory properties.

**Methods:**

The present study was aimed to determine the anti-inflammatory potential of *M*. *parviflora* leaf hydroalcoholic extract (MpHE) on AD pathology in lean and obese transgenic 5XFAD mice, a model of familial AD. The inflammatory response and Amyloid β (Aβ) plaque load in lean and obese 5XFAD mice untreated or treated with MpHE was evaluated by immunolocalization (Iba-1 and GFAP) and RT-qPCR (TNF) assays and thioflavin-S staining, respectively. Spatial learning memory was assessed by the Morris Water Maze behavioral test. Microglia phagocytosis capacity was analyzed in vivo and by ex vivo and in vitro assays, and its activation by morphological changes (phalloidin staining) and expression of CD86, Mgl1, and TREM-2 by RT-qPCR. The mechanism triggered by the MpHE was characterized in microglia primary cultures and ex vivo assays by immunoblot (PPAR-γ) and RT-qPCR (CD36) and in vivo by flow cytometry, using GW9662 (PPAR-γ inhibitor) and pioglitazone (PPAR-γ agonist). The presence of bioactive compounds in the MpHE was determined by HPLC.

**Results:**

MpHE efficiently reduced astrogliosis, the presence of insoluble Aβ peptides in the hippocampus and spatial learning impairments, of both, lean, and obese 5XFAD mice. This was accompanied by microglial cells accumulation around Aβ plaques in the cortex and the hippocampus and decreased expression of M1 inflammatory markers. Consistent with the fact that the MpHE rescued microglia phagocytic capacity via a PPAR-γ/CD36-dependent mechanism, the MpHE possess oleanolic acid and scopoletin as active phytochemicals.

**Conclusions:**

*M*. *parviflora* suppresses neuroinflammation by inhibiting microglia pro-inflammatory M1 phenotype and promoting microglia phagocytosis. Therefore, *M*. *parviflora* phytochemicals represent an alternative to prevent cognitive impairment associated with a metabolic disorder as well as an effective prophylactic candidate for AD progression.

**Electronic supplementary material:**

The online version of this article (10.1186/s12974-019-1515-3) contains supplementary material, which is available to authorized users.

## Background

Alzheimer’s Disease International Organization has predicted that 81.1 million people aged 60 years or more will be declared with dementias worldwide by the year 2040 [1]. Alzheimer disease (AD) is the main cause of dementia characterized by a progressive impairment in cognitive function (i.e., memory) and premature death [2]. Dystrophic neurites, synapses, and neuron loss result from the twisted strands of the protein tau (tangles) inside neurons and the progressive accumulation of Aβ peptides, which are produced by the consecutive proteolytic cleavage of the amyloid precursor protein (APP) by the β- and γ-secretases [3–5]. Adjacent to the amyloid core, microglial cells are frequently found, whereas astrocytes usually surround the plaques. Both cells types produce pro-inflammatory molecules like cytokines and oxygen-free radicals, among others [6]. The presence of elevated pro-inflammatory cytokine concentrations (interleukin (IL) 1 beta (IL-1β), interleukin 6 (IL-6), tumor necrosis factor (TNF)) in the AD brain has been associated with neuronal toxicity and cell death [7]. IL-1β is produced as a biologically inactive pro-form and requires to be processed by caspase-1 for its activation. Caspase-1 activity is controlled by inflammasomes, which are sensors of danger signals. The relevance of the inflammatory process in memory loss and neurodegeneration resulting from Aβ accumulation was recently shown in AD animal models lacking caspase-1 or other inflammasome components [8, 9]. Accordingly, distinct factors that play a role in the onset and progression of AD, including type II diabetes mellitus, hypertension, obesity, and dyslipidemia, have been associated to chronic inflammation [10–12]. In fact, type II diabetes is a major risk factor for dementia and has been directly associated with the Aβ accumulation in the brain, due to deficient brain Aβ clearance resulting from the competition between insulin and the Aβ for binding to the insulin-degrading enzyme [13]. Therefore, obesity and high-fat diet are apparent risk factors for dementia associated with brain insulin resistance. The excess of adipose tissue in obesity regulates adipokines (e.g., adiponectin, omentin, leptin, resistin) critical to metabolism as well as inflammatory cytokines (e.g., IL-1β, TNF, and IL-6) that correlate with insulin resistance and hyperinsulinemia [14, 15]. Thus, chronic inflammatory process in the periphery might result in dementia.

Consequently, the long-term use of non-steroidal anti-inflammatory agents in humans, such as indomethacin, sulindac, and PPAR-γ agonist, such as ibuprofen, rosiglitazone, and pioglitazone, has been directly linked with a low risk for developing AD and decreased Aβ deposition in vitro and in vivo [16, 17]. Unfortunately, the side effects produced by these drugs limit their prophylactic use. Nevertheless, there are natural products that have been widely used for a long time as a remedy for treating diseases with an inflammatory pathophysiological origin [18]. Experimental evidence indicates that polyphenolic compounds, like flavonoids, phenylpropanoids, and terpenes, among others, counteract inflammation (reviewed in [19]). Recent studies have shown that *Malva parviflora* (*M*. *parviflora*), a plant widely used in traditional medicine in America and Africa, has hypoglycemic, anti-inflammatory, and anti-oxidant effects [20, 21]. Here we evaluated the long-term effects of a *M*. *parviflora* hydroalcoholic extract (MpHE) on the development of AD pathology in obese 5XFAD mice. We show that obesity further promoted Aβ plaques formation in the 5XFAD mice. However, administration of the MpHE reversed obesity-mediated deleterious effects of Aβ deposition on memory and learning. Interestingly, this correlated with microglia-enhanced phagocytic capacity and reduced microglia M1 phenotype activation. Importantly, we demonstrated that MpHE increases microglia phagocytic activity via a PPAR-γ/CD36-dependent mechanism. The phytochemical analysis of the MpHE showed the presence of two active compounds with anti-inflammatory activity, scopoletin and oleanolic acid. Together, our results indicate that the MpHE is a promising candidate as an effective prophylactic for AD development.

## Methods

### Preparation of the *M. parviflora* hydroalcoholic extract

The leaves of *M*. *parviflora* were collected in Ozumba, state of Mexico. One specimen was deposited in the herbarium of Ethnobotanical Garden of Museum of Traditional Medicine and Herbal medicine of INAH Morelos. The species was identified by Margarita Aviles and Macrina Fuentes with the registration number 2048. Plant material was dried in dark conditions at room temperature (RT). Once dried, the plant was milled, by means of a 5HP equipment, until obtaining < 5-mm particles. Plant material (3 kg) was extracted by infusion process at 60° C with an ethanolic solution (60%). The extraction volume used was 2 L of solvent per kilogram of plant material. Liquid extract was filtered and concentrated by low-pressure distillation. Semisolid extract was finally dried by lyophilization process, obtaining 11.11% yield. This hydroalcoholic extract (HE) was submitted to liquid-liquid separation. Water and ethyl acetate phases were obtained with 100 mL of these solvents for each 5 g of the crude extract. Samples of 3 mg/mL of these fractions were used for HPLC analysis. When used for intragastric administration, aqueous suspension of the MpHE (5 mg/mL sterile water) was prepared, stored at 4° C and protected from light.

### Analysis of compounds in the hydroalcoholic extract of *M. parviflora*

Chromatographic analysis was developed by means of Waters equipment constituted by a 2695 separations module and a 2996 photodiode array detector. Separation was carried out by using an RP-18 Supersphere (Merck) column (125 × 4 mm, 5 μm). The mobile phase consisted of a gradient system with water (solvent A) and acetonitrile (solvent B). Initial conditions started with 100% of solvent A (0–1 min), followed by different proportions of solvent A:solvent B = 90:100 (2–4 min), 80:20 (5–7 min), 70:30 (8–14 min), 60:40 (15–18 min), 20:80 (19–22 min), 0:100 (23–26 min), and 100:0 (27–28 min). The sample injection volume was 10 μL with a 1 mL/min flow rate. The detection wavelength was scanned at 190–600 nm. Scopoletin quantification was achieved using a calibration curve, which was constructed by three injections of a solution prepared with a commercial reference of the coumarin standard (7.812, 15.625, 31.25, 62.5, and 125 μg/mL) (Sigma Aldrich, St Louis, MO, USA). Values of the area under the peak of chromatograms recorded at 345 nm were used to obtain the equation: *Y* = 84,221*X* − 188,219 *R*^*2*^ = 0.9995. The presence of oleanolic acid in the MpHE was established by comparison of the peak at 25.85 min with a commercial reference of this triterpene (Sigma Aldrich, St Louis, MO, USA).

### Macrophage reporter cells and NF-κB/AP-1 activation

We used a commercially available reporter macrophage cell line (RAW-Blue cells, InvivoGen, San Diego, CA, USA), which expresses the secreted embryonic alkaline phosphatase (SEAP) gene under the control of a promoter inducible by the transcription factors NF-κB and AP-1. Briefly, cells were seeded in 24-well plates in Dulbecco’s Modified Eagle Medium (DMEM), 4.5 g/l glucose, 10% heat-inactivated FBS (30 min at 56 °C), 2 mM L-glutamine, 100 mg/mL Normocin, and 200 mg/mL Zeocin. Then, cells were treated with lipopolysaccharide (LPS; 100 ng/mL) alone or together with three different concentrations of MpHE (0.1, 0.5, and 1.0 mg/mL), oleanolic acid (15, 26, 40 μg/mL) or scopoletin (3, 8, 24 μg/mL) for 12 h. In addition, cells were pre-treated with LPS or MpHE for 30 min, and then MpHE or LPS was added for 12 h. NF-κB and AP-1 activity was evaluated indirectly by quantifying the SEAP activity in the culture supernatants. Briefly, 50 μL from each sample were added to 150 μL of the QUANTI-Blue assay buffer (InvivoGen, San Diego, CA, USA) and incubated at 37 °C for 30 min. PBS or the used vehicle to dissolve oleanolic acid or scopoletin were used as negative control. Absorbance at 650-nm wavelength was measured and the SEAP activity was calculated as fold change for each sample (SEAP activity in medium from treated cells minus background (medium without cells) over SEAP activity in medium from untreated cells minus background). In parallel, trypan blue staining was used to determine the cell viability after MpHE treatment.

### Animals

The 5XFAD transgenic mice, expressing the human APP gene with three familial AD mutations (Swedish (K670N, M671L), Florida (I716V), and London (V717I)) and the human presenilin 1 carrying two mutations (M146L and L286V) were obtained from The Jackson Laboratory (Bar Harbor, ME, USA). Mice were housed in groups of five per cage, in a temperature- (22 ± 1 °C) and humidity-controlled room, with a 12-h light/dark cycle. Food and water were available ad libitum. All tests were performed during the light cycle. Male 5XFAD mice and male wild type (Wt) littermates were used (C57BL/SJL genetic background). Transgenic and Wt mice (6 weeks old) were fed with normal diet (ND) (caloric percentage, 18%, 3.1 kcal/g; Teklad, Harlan Laboratories), or with a high-fat diet (HFD) (caloric percentage, 60%, 5.24 kcal/g; Research diets. Inc, USA). These groups of mice received 50 mg/kg/day of the MpHE or water (vehicle) intragastrically during 8 months. Two additional groups from Wt ND and Wt ND treated with MpHE (C57BL genetic background) were included to determine spatial learning.

For the dose-response experiments, a LPS-mediated neuroinflammation model was used. CD1 male mice were intraperitoneally injected with 125 μg/kg of LPS or vehicle as control for 7 consecutive days. LPS injected mice then received 25, 50, or 100 mg/kg/day of the MpHE or water (vehicle) intragastrically during 7 days. Spatial learning and memory were determined. The Institutional Bioethical Committee approved all animal experiments described in this study.

### Metabolic analysis

Food intake and body weight were measured weekly. After 7 months of treatment, glucose tolerance test (GTT) was performed after 6 h food deprivation by intraperitoneal (i.p.) glucose administration (1.8 g/kg body weight). Blood glucose concentrations were measured before glucose administration (*t* = 0) and 15, 30, 60, and 120 min after injection. One month after, insulin resistance test (IRT) was performed after 6 h food deprivation by i.p. injection of insulin (Humulin® R 1 mU/gram of body mass). Blood glucose concentrations were measured before insulin administration (*t* = 0) and 15, 30, 60, and 120 min after insulin administration using a Glucometer (Accucheck; Roche) and the data were reported as milligrams per deciliter. Area under the curve (AUC) for glucose concentration in both cases was calculated as reported previously [22].

### Immunofluorescence

Mice were anesthetized with ketamine/xylazine (90 mg/kg/9 mg/kg, i.p.) and perfused transcardially with isotonic saline followed by 4% paraformaldehyde (PFA) in phosphate buffer saline (PBS; pH 7.4). Brains were post-fixed in 4% PFA overnight, followed by 30% (*w*/*v*) sucrose solution at 4 °C. Immunofluorescence was performed by the free-floating antibody staining method, using 30-μm-thick sagittal cryostat slices. The slices were washed three times with PBS-T (0.25% Triton X-100 in PBS) for 5 min each and incubated in blocking buffer (PBS-T 0.1% bovine serum albumin) for 1 h at RT. Then, the slices were incubated with mouse monoclonal antibody against Glial fibrillary acidic protein (GFAP) (AB_561049; 1:250) overnight at 4 °C, washed with PBS-T two times for 5 min each and incubated with secondary antibody goat anti-mouse Alexa Flour 488 (AB_2534069; 1:400) in blocking buffer for 2 h at RT. After 2 washes with PBS-T, free-floating brain slices were then mounted with Fluoroshield (Sigma Aldrich Inc) and sealed with a coverslip. Additionally, goat antibody anti-Iba1 (AB_2224402; 1:200) and the secondary antibody donkey anti-goat Alexa Fluor 568 (AB_175704; 1:200, Abcam Inc) were used to identify microglial cells in the brain sections. Images were captured using the Zeiss Axioskop Observer Z1 inverted fluorescence microscope.

### Thioflavin-S staining

PFA-fixed brain tissues of each group were stained in 1% aqueous Thioflavin-S for 5 min. After this, slices were washed sequentially in 80% ethanol, 70% ethanol, and distilled water, then dried and mounted as above described. The brain slices stained with Thioflavin-S were analyzed in a Zeiss Axioskop microscope using an emission spectrum of 550 nm. Images were captured using the Zeiss Axioskop fluorescence microscope. Two or three slices were analyzed in four animals/group. Analyses were performed across the dentate gyrus using a × 5 objective and the automated target detection mode from Image J software (National Institutes of Health, Bethesda, MD, USA). Image size was 1800 × 1800 μm and the percentage of load area was calculated (load area/total area). Additionally, to determine the fraction of amyloid plaques overlapping with microglia in the hippocampus and cortex from 5XFAD mice administered with vehicle or MpHE, the Manders’s coefficient was determined using the plugin JacoP [23] in Image J software (National Institutes of Health, Bethesda, MD, USA).

### Behavioral assessment of cognitive functions by Morris water maze test

Spatial learning memory was assessed by the Morris Water Maze behavioral test. Mice were tested in a 1.83 m circular pool filled with water mixed with nontoxic white paint (titanium oxide). The tank was divided into four quadrants (NW, SW, SE, and NE) of equal size. Mice were trained to mount a hidden/submerged (1.0 cm below water surface) escape platform (15-cm diameter) in a restricted region of the pool (NW). Mice were trained for 5 consecutive days from the four quadrants of the tank; they were placed into the water facing the wall and were allowed to swim for a maximum of 90 s. The trial ended when a mouse climbed onto the platform or after the 90-s interval had elapsed. If a mouse did not locate the platform during a trial, it was placed on the platform by the experimenter during 20 s, and then moved to a holding cage. Escape latencies to the hide platform during the learning phase were analyzed using the integrated time traveled and time needed to reach the platform (AUC) for each group. After two days, a memory test was performed in absence of the platform. Mice were released from the four start points to the platform location and allowed to swim freely in the pool for 90 s. Escape latency and swimming paths were recorded.

### Microglial cell culture

Primary cultures of microglial cells were prepared from neonatal (postnatal days 1-3) CD1 mice or from 8- and 10-months-old Wt and 5XFAD mice. Briefly, neonatal brain tissues were mechanically dissociated, treated with Trypsin (0.25 mg/mL, Sigma Aldrich Inc) and DNase I (0.28 mg/mL) at 37 °C for 30 min. After extensive washing, dispersed brain cells were plated as mixed glia cultures (enriched with microglial and astroglia cells) at the density of two brains per dish in DMEM/F12 medium supplemented with 10% FBS, 2 mM glutamine, and antibiotics onto 100-mm Petri dishes at 37 °C and 5% CO_2_. Eight days after plating, the microglia cells were separated from astrocytes by shaking for 5 h at 250 rpm at RT. The neonatal microglial cultures purity was analyzed by flow cytometry, RT-PCR, and immunofluorescence assays using specific markers for microglia (F4/80), CD11b, astrocytes (GFAP), and neurons (neurofilament M). The antibody anti-F4/80 (AB_1140040; 1.200), anti-CD11b (AB_389305; 1:2000), and anti-GFAP (AB_561049; 1:200) were used for this purpose.

Alternatively, adult brain tissue from wild type and 5XFAD mice was mechanically dissociated and the Neural Tissue Dissociation Kit (Miltenyi Biotec, USA) was used to obtain single-cell suspension. The myelin debris was removed with the Myelin Removal Beads II (Miltenyi Biotec, USA), and the microglia were isolated by positive selection using the CD11b (microglia) microbeads (Miltenyi Biotec, USA), according to the manufacturer’s instructions. The adult microglial cultures purity was analyzed by flow cytometry assays using anti-CD11b antibody (AB_389305; 1:2000).

### Microglia classical activation

Neonatal primary microglial cultures (1 × 10^5^ cells per well) were plated onto Lab-Tek coverglass chamber wells (Nalge Nunc International, Rochester, NY, USA). After 1 day, the cells were stimulated with 100 ng/mL LPS in the presence or absence of MpHE (1 mg/mL) for 24 h. Control cells were treated with PBS or MpHE alone. Subsequently, the cells were fixed in 4% PFA for 30 min at RT and permeabilized with PBS containing 0.1% Triton TX-100 for 30 min at RT. F-actin was visualized by incubating cells for 1 h in the dark with Alexa Fluor 488-conjugated phalloidin (AB_2315147; 1:500). Cell nuclei were labeled with 4′, 6-diamidino-2-phenylindole (DAPI, 1:5000, Molecular probes) for 15 min. Microglial cells were imaged using the Olympus FluoView 1000 confocal multiphoton microscope. The different microglial cell morphologies (amoeboid, ramified, and intermediate shapes) were quantified in each treatment from seven random fields per group. The length of the protrusions of the microglial cells was determined using Neurite Tracer plugin from ImageJ software (National Institutes of Health, Bethesda, MD).

### In vitro phagocytosis assay

Eight days after separating astroglial cells, microglia from eight and 10-months-old Wt and 5XFAD mice were plated onto Lab-Tek coverglass chamber wells (1 × 10^5^ cells per well) (Nalgene Nunc International, Rochester, NY, USA) and treated with MpHE (1 mg/mL) for 24 h. The medium was then replaced with fresh medium containing 1 × 10^6^ fluorescent *E*. *coli* bacteria [24] or unlabeled latex beads (0.8 μm diameter) (Calibrite, Becton Dickinson), and incubated for 1 or 4 h, respectively. The medium was removed, the microglial cells were washed extensively with PBS, treated with Geneticin (20 μg/mL) for 30 min, and fixed in 4% PFA (Sigma Aldrich Inc) for 30 min at RT. Then, cells were permeabilized with PBS containing 0.1% Triton TX-100 for 30 min at RT. Actin and the nucleus were visualized as described above. The percentage of cells capable of internalizing *E*. *coli* bacteria and the average of *E*. *coli* ingested by cells were determined. The phagocytosis index was determined by multiplying the percentage of microglial cells with internalized bacteria by the average number of internalized *E*. *coli* bacteria [25] or unlabeled latex beads for each sample. Data were collected from seven random fields per group. Microglial cells were imaged using the Olympus FluoView 1000 confocal multiphoton microscope.

### PPAR-γ protein levels and ex vivo phagocytosis assay

Neonatal primary microglial cultures (2.5 × 10^5^ cells) were left untreated or pre-treated with 100 μM GW9662, a specific PPAR-γ inhibitor. After 1 h, cultures were treated with MpHE (0.1 and 1 mg/mL) or with pioglitazone (10 μM; a PPAR-γ agonist) or with oleanolic acid (4.5 μg/mL and 45 μg/mL) for 24 h. The PPAR-γ levels from the cell extracts were determined by immunoblot. Additionally, PPAR-γ levels were also determined in the frontal cortex cell extracts of 5XFAD and Wt mice fed with either ND or HFD non-treated (vehicle) or treated with MpHE.

Ex vivo Aβ phagocytosis assays were performed as previously described [26]. Briefly, adult 5XFAD transgenic mice were perfused with saline solution. Brains were removed and snap frozen in dry ice. After, 10-μm-thick sagittal cryostat slices were obtained and mounted onto poly-D-lysine-coated glass coverslips. Brain slices were dried for at least 2 h at RT and then washed twice with PBS and DMEM serum-free media (Gibco). Each brain slice was cultured with neonatal primary microglial cultures (2.5 × 10^5^ cells) previously untreated or treated with MpHE (1 mg/mL) for 12 h or 100 μM of GW9662 (Sigma Aldrich) for 1 h in FBS-free DMEM medium. After 12 h, the cells were washed and the medium was replaced by DMEM containing 5% FBS and 20% L929 supernatant for 24 h at 37 °C with 5% CO_2_. Sections were then washed twice with PBS and fixed with 4% PFA for subsequent histology. The brain slices were stained with Thioflavin-S and the plaque’s number and size were quantified.

### In vivo phagocytosis assay

Male 5XFAD mice and male Wt littermates were used (C57BL/SJL genetic background). Transgenic and Wt mice (8 weeks old) received 50 mg/kg/day of the MpHE or water (vehicle) intragastrically during 2 months. Then, mice were intraperitoneally injected with the PPARγ inhibitor GW9662 (5 mg/kg) or vehicle (5% DMSO/95%PBS) for the last 3 days before sacrifice. For the in vivo Aβ phagocytosis assays, mice were intraperitoneally injected with 10 mg/kg methoxy-X04 (Tocris) 6 h before sacrifice. Brains were removed and cell suspension was obtained by enzymatic (0.2 mg/mL papain) and mechanical dissociation. Myelin debris was removed by centrifugation (10 min at 700 g) using 30% Percoll (Sigma). The cells present in the pellet were suspended, immunolabeled for microglia specific markers using the antibody anti-CD11b (AB_389305; 1:2000) and the anti-CD36 (AB_2072639;1:200) and analyzed by flow cytometry using a FACSCanto II (BD Biosciences). The CD36 expression analysis was done on the CD11b^+^ cells while the methoxy-X04 staining analysis was performed on the CD11b^+^/CD36^+^ population. Wt unstained and methoxy-X04 stained cells were used to determine background fluorescence and the threshold for non-phagocytic cells, respectively.

### PCR and qPCR

Total RNA was isolated from primary cultures (neonatal microglia) or from tissue (cortex) as previously described [27]. Reverse transcription was performed using M-MLV reverse transcriptase (Invitrogen, ThermoFisher Scientific) and oligo (dT) primer. Neonatal microglial cultures purity was determined by semi-quantitative PCR using the following forward (F) and reverse (R) primers: For F4/80: F 5′-GTG CCA TCA TTG CGG GAT TC-3′ and R 5′-GAC GGT TGA GCA GAC AGT GA-3′, for Neurofilament-M: F 5′-GAA CCA CGA GAA GGC TCA AG-3′ and R 5′- CCT CCT CTT CGT GAT TGC TC-3′, for GFAP: F 5′-CAG TTC AGC TGC CAG CGC CT-3′ and R 5′-GCA GAG GCA GGG CAG GAT GG-3′, and for Actin: F 5′-GGG TCA GAA GGA CTC CTA TG-3′ and R 5′-GGT CTC AAA CAT GAT CTG GG -3′. Quantitative real time PCR (qPCR) was carried out on ABI PRISM 7500 sequence detection system (Applied Biosystems, Foster City, CA, USA) using cDNA from Wt or 5XFAD lean or obese mice cortex treated or not treated with MpHE in combination with Maxima SYBR Green/ROX qPCR Master Mix (ThermoFisher Scientific) and specific primers. For TREM-2: F 5′-CTG GAA CCG TCA CCA TCA CTC-3′, and R 5′-CGA AAC TCG ATG ACT CCT CGG-3′; for Mgl1: F 5′-TGA GAA AGG CTT TAA GAA CTG GG-3′, and R 5′-GAC CAC CTG TAG TGA TGT GGG-3′; for CD86: F 5′-GAC CGT TGT GTG TGT TCT GG-3′, and R 5′-GAT GAG CAG CAT CAC AAG GA-3′; for TNF: F 5′-CAG GCG GTG CCT ATG TCT C-3′, and R 5′- CGA TCA CCC CGA AGT TCA GTA G-3′; for CD36: F 5′-GAG CAA CTG GTG GAT GGT TT-3′, and R 5′-GCA GCA GAA TCA AGG GAG AG-3′; for GAPDH: F 5′-GGG AAG CTC ACT GGC ATG G 3′, and R 5′-CTT CTT GAT GTC ATC ATA CTT GGC AG-3′; and for Ndufa-10 (UBC): F 5′-CCG CCT TCT TCA GTA TGC AGA-3′, and R 5′- TGC TTT CGG ATA TAG CCC TGG. Relative expression was calculated by normalizing to Ndufa-10 or GAPDH using the 2^−∆∆CT^ method [28].

### Western blotting

Neonatal microglial culture homogenates were obtained by scraping with ice-cold lysis buffer (20 mM Tris pH 7.4, 137 mM NaCl, 2 mM PPiNa, 2 mM EDTA, 1% Triton X-100, 10% glycerol, 0.5 mM DTT, 25 mM β-glycerophosphate, 200 mM Na_3_VO_4_, 1 mM PMSF, and complete protease inhibitor (Roche)). Lysates were incubated 10 min at 4 °C, then centrifuged at 14,000*×g* for 15 min at 4 °C, and stored at − 70 °C until use. Protein concentration was determined using the Bradford reagent (Bio-Rad). Total cell extracts (20 μg) were resolved by SDS-PAGE and transferred onto nitrocellulose membranes (GE Healthcare). Membranes were blocked with 5% BSA in Tris-buffer saline (TBS, 10 mM Tris pH 7.5, 150 mM NaCl) with 0.1% Tween-20 (Sigma) (TBS-T) and incubated with the primary antibody anti-PPAR-γ (AB_2283990; 1:5000, Santa Cruz Biotechnology), anti-actin (AB_2223041; 1:8000, Millipore), or anti-GAPDH (AB_10622025; 1:6000, Cell Signaling) overnight at 4 °C. After three washes with TBS-T, membranes were incubated with secondary antibody anti-rabbit-HRP (1:10000).

### ELISA

Microglia from 8-month-old Wt and 5XFAD mice were plated onto Lab-Tek coverglass chamber wells (1 × 10^5^ cells per well) (Nalgene Nunc International, Rochester, NY). After one day, cells were stimulated with 100 ng/mL LPS in the presence or absence of MpHE (1 mg/mL) for 24 h. Control cells were treated with PBS or MpHE alone. TNF and IL-6 levels were quantified from the primary microglial cultures supernatants using a commercial ELISA Kit (Biolegend, Cat. 430904 and 431304) following the manufacturer’s instructions.

### Adipose tissue histochemistry

Adipose tissue was fixed in 10% paraformaldehyde and embedded in paraffin. 5-μM-thick sections were prepared, deparaffinized in xylene, rehydrated in a graded ethanol series, and then stained with hematoxylin and eosin. Analysis of adipocyte histology was performed using the ImageJ software according to the manual procedure (http://rsbweb.nih.gov/ij/). Immune cells infiltration in adipose tissue was quantified by calculating the ratio of infiltration on 10 fields (× 40), of 3 slides for each individual mice, using 3 mice for each group. Light microscopic images were acquired using a Zeiss LSM510/UV Axiovert 200M confocal microscope with a Nikon COOLPIX 5000 color camera.

### Statistical analysis

Results presented correspond to at least three independent experiments. No sample size calculation was performed. Data are shown as the mean ± SEM or SD. Data were analyzed by one-way ANOVA followed by Tukey’s post hoc test, two-way ANOVA followed by Dunnet’s or Bonferroni’s multiple comparison test, and three-way ANOVA followed by Tukey’s multiple comparison test. Differences were considered statistically significant with a *p* value < 0.05. Statistical analyses were performed in Prism 7.0b or Prism 8.0 (Graphpad Software, Inc.).

## Results

### *M. parviflora* hydroalcoholic extract inhibits LPS-induced NF-κB activity in mouse RAW-Blue macrophages

**Fig. 1:**
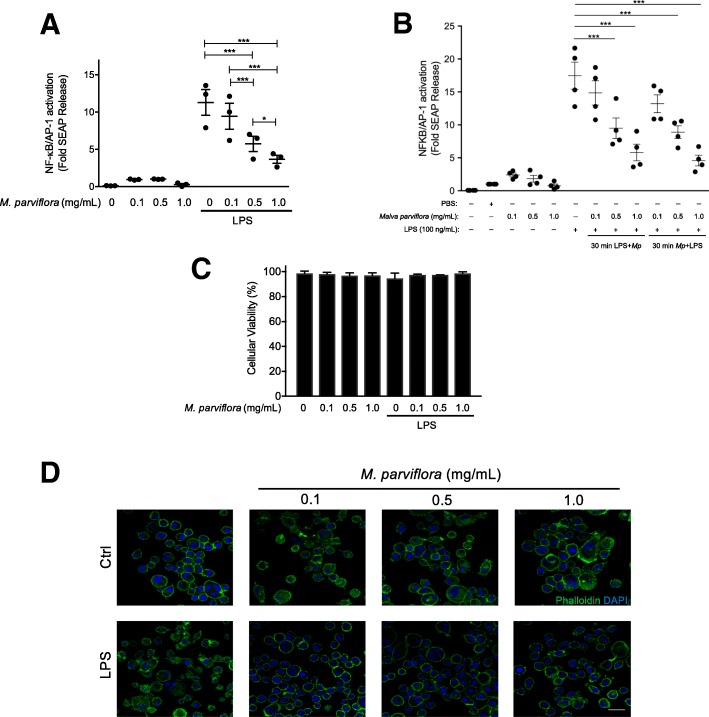
The *Malva parviflora* hydroalcoholic extract inhibits LPS-induced NF-κB activity in mouse RAW-Blue macrophages. **a** RAW-Blue macrophages were untreated or treated with LPS (100 ng/mL) in the presence of the indicated concentrations of *M*. *parviflora* hydroalcoholic extract (HE) and 12 h later embryonic alkaline phosphatase (SEAP) activity (driven by NF-κB/AP-1 activation) was determined in the supernatants as described in the “Methods” section. Values are expressed as fold increase relative to SEAP reporter activity in untreated control cells. **b** RAW-Blue macrophages were pre-treated with LPS or *M*. *parviflora* for 30 min and 12 h later embryonic alkaline phosphatase (SEAP) activity (driven by NF-κB/AP-1 activation) was determined in the supernatants as described in the “Methods” section. Data are shown as mean ± SEM. Statistical analysis was performed by two-way ANOVA with repeated measures followed by post hoc Sidak’s multiple comparisons test. This analysis revealed a significant effect for the MpHE concentration *F*(3,12) = 28.9, *p < 0*.*001*; for the LPS administration *F*(1,4) = 31.49 *p = 0*.*005* and for the *M*. *parviflora* and LPS interaction *F*(3,12) = 30.88, *p < 0*.*001*. (***p < 0.*001*, *p = 0.*03*). **c** Cellular viability of RAW-Blue macrophages was evaluated using trypan blue exclusion after exposure to the MpHE at three different concentrations (0.1, 0.5, and 1.0 mg/mL) for 12 h in presence or absence of LPS (100 ng/mL). **d** RAW-Blue cells were treated with the indicated amount of the *M*. *parviflora* in the absence (Ctrl) or in the presence of LPS (100 ng/mL) for 12 h. Cells were fixed and stained with phalloidin and DAPI for F-actin (green) and nuclei (blue) detection, respectively. Images were captured using the Olympus FluoView 1000 confocal multiphoton microscope (Scale bar, 30 μm)

Previous studies have demonstrated the anti-inflammatory and anti-oxidant effects of *M*. *parviflora* [21]. Therefore, we determined whether the MpHE negatively regulates LPS-induced NF-κB transcriptional activity as an indication of its anti-inflammatory activity in RAW-blue macrophage cells exposed to LPS. To this end, three different concentrations of MpHE (0.1, 0.5, and 1.0 mg/mL) together with LPS (100 ng/mL) were applied to RAW-Blue cells for 12 h. The MpHE inhibited NF-κB-driven SEAP activity by LPS in a concentration-dependent manner (*F* (3, 12) = 28.9; 0.5 mg/mL *M*. *parviflora*, *p* < 0.001, and 1 mg/mL *M*. *parviflora*, *p* < 0.001 compared with the LPS treatment and with the LPS plus 0.1 mg/mL *M*. *parviflora* group) (Fig. 1a). Further, we evaluated the therapeutic potential of MpHE by pretreating the cells with LPS for 30 min and then MpHE was added for 12 h. As for the preventive effect, MpHE reverted the NF-κB-driven SEAP activity by LPS in a concentration-dependent manner (*F* (3,12) 27.92; 0.5 mg/mL *M*. *parviflora*, *p* < 0.001 and 1 mg/mL *M*. *parviflora*, *p* < 0.001 compared with the LPS treatment and with the LPS plus 0.1 mg/mL *M*. *parviflora* group) (Fig. 1b). Similarly, cell exposure to MpHE first and after 30 min to LPS, exhibited a clear inhibition of NF-κB-driven SEAP activity by LPS in a concentration-dependent manner (*F* (3,12) 27.92; 0.5 mg/mL *M*. *parviflora*, *p* < 0.001 and 1 mg/mL *M*. *parviflora*, *p* < 0.001 compared with the LPS treatment and with the LPS plus 0.1 mg/mL *M*. *parviflora* group) (Fig. 1b). Importantly, under these experimental conditions, no changes in cell viability or morphology were observed (Fig. 1c, d). Altogether these data confirm the anti-inflammatory effect of the MpHE without cytotoxic effect.

### *M. parviflora* hydroalcoholic extract improves glucose homeostasis in 5XFAD mice fed with a high fat diet

**Fig. 2:**
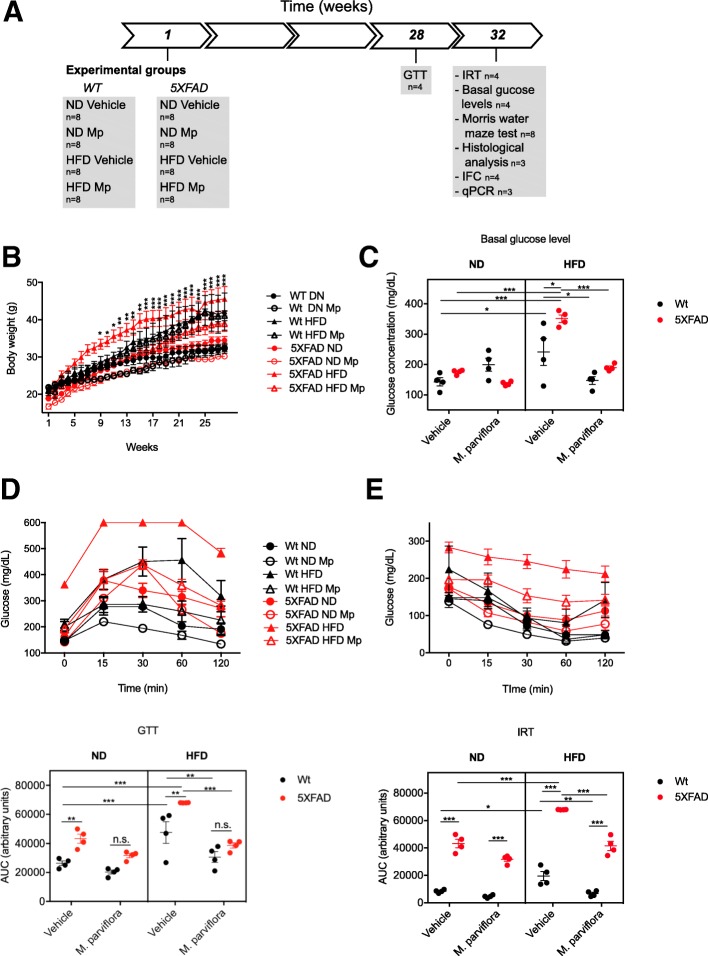
*Malva parviflora* hydroalcoholic extract promotes insulin sensitivity and glucose tolerance in 5XFAD transgenic mice fed with high-fat diet. **a** Graphical time line of the study design and experimental procedures. GTT, glucose tolerance test; IRT, insulin resistant test; IFC, immunofluorescence. **b** Body weight gain (g) was measured weekly during 28 weeks in wild type (Wt) mice fed with normal diet (ND; closed black circle) or high-fat diet (HFD; closed black triangle) and those that received intragastrically water or 50 mg/kg/day of the MpHE (Mp): Wt ND + Mp (open black circle) or Wt HFD + Mp (open black triangle), or transgenic 5XFAD mice fed with a normal diet (ND; closed red circle) or a high-fat diet (HFD; closed red triangle) that received intragastrically water or 50 mg/kg/day of the MpHE (Mp): 5XFAD ND + Mp (open red circle), or 5XFAD HFD + Mp (open red triangle). Data are shown as mean ± SEM, *n* = 4 in Wt ND, *n* = 4 in Wt HFD, *n* = 4 in Wt ND + Mp, *n* = 4 in Wt HFD + Mp, *n* = 10 in 5XFAD ND, *n* = 10 in 5XFAD HDF, *n* = 10 in 5XFAD ND + Mp and *n* = 9 in 5XFAD HFD + Mp. Statistical analysis was performed by two-way ANOVA with repeated measures followed by post hoc Bonferroni’s multiple comparisons test. This analysis revealed a significant effect for the genotype *F*(7,47) = 9.971, *p < 0*.*001*; for the time *F*(27,1269) = 206.5 *p < 0*.*001* and for the genotype and time interaction *F*(189, 1269), *p < 0*.*001*. (*p = 0.*0488*, **p = 0.*0069*, ***p < 0.*001*). **c** Basal blood glucose concentrations in Wt or transgenic 5XFAD mice after 8 months of being fed with ND or HFD alone or treated with Mp. Data are shown as mean ± SEM, *n* = 4 for all the groups. Statistical analysis was performed by three-way ANOVA followed by Tukey’s multiple comparisons test. This analysis revealed a significant effect for the genotype *F*(1,24) = 5.029, *p = 0*.*03440*; for the diet *F*(1,24) = 25.08, *p < 0*.*001*; for the *M*. *parviflora* treatment *F*(1,24) = 18.56; *p = 0*.*002*; for the genotype and diet interaction *F*(1,24) = 11, *p = 0*.*0029*; for the *M*. *parviflora* treatment and diet interaction *F*(1,24) = 24.99, *p < 0*.*001*; for the genotype and *M*. *parviflora* treatment interaction *F*(1,24) = 8.661 *p = 0*.*0071*; and for the genotype, *M*. *parviflora* treatment, and diet interaction *F*(1,24) = 0.2458, *p = 0*.*6245*. **d** Blood glucose during intraperitoneal glucose tolerance test (GTT) of Wt or transgenic 5XFAD mice 8 months after of being fed with ND or HFD alone or treated with *M*. *parviflora* (Mp). Bar graph (lower panel) represents the area under the curve (AUC). Data are shown as mean ± SEM, *n* = 4 for all the groups. Statistical analysis was performed by three-way ANOVA followed by Tukey’s multiple comparisons test. This analysis revealed a significant effect for the genotype *F*(1,24) = 36.52, *p < 0*.*001*; for the diet *F*(1,24) = 45.37, *p < 0*.*001*; for the *M*. *parviflora* treatment *F*(1,24) = 46.23, *p < 0*.*001*; for the genotype and diet interaction *F*(1,24) = 0.0002756, *p = 0*.*9869*; for the *M*. *parviflora* treatment and diet interaction *F*(1,24) = 9.277, *p = 0*.*0056*; for the genotype and *M*. *parviflora* treatment interaction *F*(1,24) = 3.678 *p = 0*.*0671*; and for the genotype, *M*. *parviflora* treatment, and diet interaction *F*(1,24) = 0.5456, *p = 0*.*4673*. **e** Blood glucose levels were measured at several time points following insulin administration during the insulin resistance test (IRT) of Wt or transgenic 5XFAD mice 8 months after of being fed with ND or HFD alone or treated with *M*. *parviflora* (Mp). Bar graph (lower panel) represents the area under the curve (AUC). Statistical analysis was performed by three-way ANOVA followed by Tukey’s multiple comparisons test. This analysis revealed a significant effect for the genotype *F*(1,24) = 356.6, *p < 0*.*001*; for the diet *F*(1,24) = 38.47, *p < 0*.*001*; for the *M*. *parviflora* treatment *F*(1,24) = 36.14, *p < 0*.*001*; for the genotype and diet interaction *F*(1,24) = 7.772, *p = 0*.*0102*; for the *M*. *parviflora* treatment and diet interaction *F*(1,24) = 4.078, *p = 0*.*0547*; for the genotype and *M*. *parviflora* treatment interaction *F*(1,24) = 15.14 *p = 0*.*007*; and for the genotype, *M*. *parviflora* treatment, and diet interaction *F*(1,24) = 3.468, *p = 0*.*0749*

Given that alteration in glucose homeostasis resulting from obesity exacerbates Aβ-induced memory loss [29] and that chronic inflammation is key for AD development [30], we evaluated whether long-term administration (8 months) of the MpHE could attenuate the effects of an altered glucose metabolism on the development of the pathological changes associated with AD (Fig. 2a). The MpHE concentration used for the in vivo experiments was chosen based on our studies where increasing concentrations (25, 50 and 100 mg/Kg) of MpHE protected from learning and memory deficit in a LPS-mediated neuroinflammation mouse model (Additional file 1: Figure S1). Since the three concentrations tested under our experimental conditions were equally protective we administrated 50 mg/kg/day of the MpHE to the 5XFAD transgenic and Wt mice, to ensure an effect and to avoid possible side effects on the animal health by the long-term administration of the higher dose. First, we evaluated glucose metabolism in obese 5XFAD mice. From the fifth week after HFD administration, the 5XFAD mice increased significantly (*p = 0*.*0238*) their body weight compared to 5XFAD mice fed with ND, reaching the maximum difference (*p* < 0.001) from week 15 onwards (Fig. 2b). After 7 months, the glucose baseline in the 5XFAD mice fed with HFD was approximately 375 mg/dL while in the mice fed with ND was 176.8 mg/dL (*p <* 0.001) (Fig. 2c). Consistent with this, glucose tolerance was significantly impaired in obese 5XFAD mice. At 15, 30, 60, or 120 min after glucose load, blood glucose levels in the HFD group were 1.6, 1.8, 1.9, or 1.8 times higher than the glucose levels in the 5XFAD mice fed with ND (Fig. 2d, upper panel). Similarly, the AUC value for the HFD-fed 5XFAD mice was significantly higher than those fed with ND (*p < 0*.*001*) (Fig. 2d, lower panel). In accordance with this, the insulin sensitivity was decreased in the 5XFAD mice consuming HFD (Fig. 2e). At 15 min after insulin administration, blood glucose levels decreased by 25% in ND-fed group and only by 11% in HFD-fed group, as compared to glucose levels before insulin injections. At 30 min after insulin administration, the blood glucose levels were decreased by 45% in ND-fed 5XFAD mice and by 17% in HFD-fed group (Fig. 2e, upper panel). The AUC value for the HFD-fed 5XFAD mice was significantly higher (*p* < 0.001) than those 5XFAD mice fed with the ND (Fig. 2e, lower panel). These results confirm that 5XFAD mice fed with the HFD present insulin resistance and glucose intolerance. In parallel, HFD was administered to wild type mice for 8 months. As expected, glucose intolerance (*p =* 0.009) and insulin resistance (*p =* 0.0182) were observed in response to HFD administration in the wild type animals compared with those ND-fed wild type mice. Significant difference in the gain body weight was observed from week 22 (*p = 0*.*0236*). Furthermore, MpHE treatment improved glucose tolerance (*p =* 0.0268) and insulin sensitivity (*p =* 0.0045) in the wild type mice fed with the HFD (Fig. 2d, e). While in wild type mice fed with the ND, the MpHE had a marginal effect on glucose tolerance and insulin sensitivity.

The treatment with the MpHE delayed the increase in body weight in the 5XFAD animals fed with HFD observing significant difference since the seventh week (*p = 0*.*0267*) between both groups (Fig. 2b). Accordingly, with previously published data indicating that *M*. *parviflora* regulates glucose metabolism in a type 1 diabetes animal model [20], no significant differences were observed between animals fed with HFD + *M*. *parviflora* extract respect to ND-fed animals at all time points assayed (*p =* 0.4719 at the week 28) (Fig. 2b). After eight months of HFD feeding and MpHE treatment, the glucose baseline levels (Fig. 2c), the glucose tolerance (Fig. 2d), and insulin sensitivity (Fig. 2e) were similar to those transgenic mice fed with ND. Moreover, in the case of insulin resistance test, the AUC value obtained in HFD transgenic mice treated with MpHE was twofold lower than in the HFD transgenic group without treatment (*p <* 0.001) (Fig. 2e, lower panel). These results showed that MpHE prevented the systemic insulin resistance and glucose intolerance produced by HFD in the 5XFAD mice.

### *M. parviflora* hydroalcoholic extract reduces amyloidosis and astrogliosis in the 5XFAD mice fed with a high fat diet

**Fig. 3:**
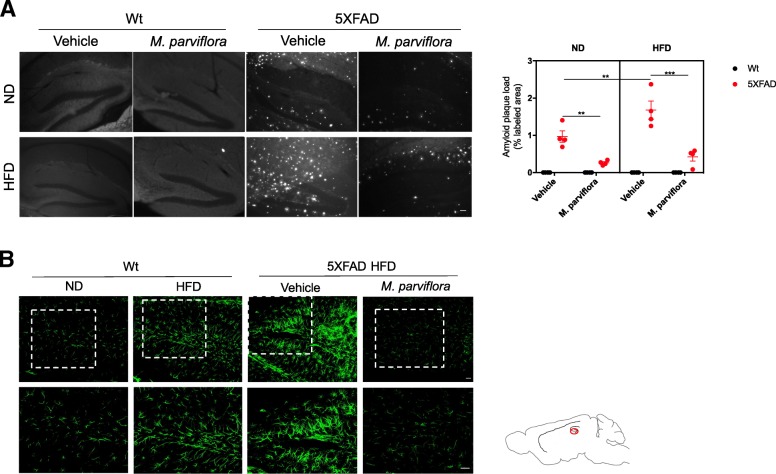
*Malva parviflora* hydroalcoholic extract reduces amyloid plaque formation and astrogliosis in 5XFAD transgenic mice fed with high-fat diet. **a** (Left) Representative micrographs of amyloid plaques labeled with thioflavin S in the hippocampus of wild type (Wt) or 5XFAD mice fed with either normal diet (ND) or high-fat diet (HFD) non-treated (Vehicle) or treated with *Malva parviflora* hydroalcoholic extract (*M*. *parviflora*) for 8 months (scale bar 100 μm). (Right) Graph represents amyloid plaque loads in hippocampus of 5XFAD transgenic mice fed with normal diet (ND) or high-fat diet (HFD), treated with vehicle or *M*. *parviflora* extract. Data are shown as mean ± SEM, *n* = 4 animals per group. Statistical analysis was performed by three-way ANOVA followed by Tukey’s multiple comparisons test. This analysis revealed a significant effect for the genotype *F*(1,24) = 113.7, *p < 0*.*001*; for the diet *F*(1,24) = 7.781, *p = 0*.*01*; for the *M*. *parviflora* treatment *F*(1,24) = 39.54, *p < 0*.*001*; for the genotype and diet interaction *F*(1,24) = 7.781, *p = 0*.*01*; for the *M*. *parviflora* treatment and diet interaction *F*(1,24) = 3.03 *p = 0*.*094*; for the genotype and *M*. *parviflora* treatment interaction *F*(1,24) = 39.54 *p < 0*.*001*; and for the genotype, *M*. *parviflora* treatment, and diet interaction *F*(1,24) = 3.03, *p = 0*.*094*. **b** Astrogliosis was evaluated by immunofluorescence on hippocampal slides (dentate gyrus) from Wt and 5XFAD mice fed with high-fat diet (HFD) non-treated (vehicle) or treated with *M*. *parviflora* extract for 8 months. Astrocytes were imaged using the Olympus FluoView 1000 confocal multiphoton microscope (× 40, scale bar 30 μm). Lower row represents a × 2 digital magnification from boxed areas (scale bar 30 μm). Sketch indicates the analyzed area within the hippocampus

To determine whether MpHA attenuates the negative effects of altered glucose metabolism resulting from obesity on memory loss induced by Aβ accumulation, first, we evaluated the accumulation of extracellular amyloid plaques in the hippocampus, a hallmark in the AD pathology (Fig. 3a). Amyloid plaques were significantly increased in the 5XFAD mice after 8 months of HFD feeding compared with 5XFAD mice fed with the ND (*p* < 0.0029) (Fig. 3a, right panel). As reported [31], this result confirms that HFD aggravates the AD pathology. However, long-term administration of MpHE reduced Aβ accumulation throughout hippocampus of 5XFAD mice fed either with ND (*p =* 0.0028) or with HFD (*p* < 0.001) compared to the Aβ levels observed in animals administered with the vehicle (Fig. 3a, right panel).

Astrocyte activation (astrogliosis) in response to Aβ accumulation is a characteristic of AD development. GFAP staining of sagittal sections from 9-months-old 5XFAD mice showed that long-term treatment (8 months) with the MpHE reduced the astrogliosis throughout the hippocampus of HFD-fed 5XFAD mice (Fig. 3b).

Together, these results point out that MpHE attenuates the inflammatory process in the brain triggered from Aβ accumulation resulting from APP and Presenilin 1 mutations and that MpHE also attenuates the effect of altered glucose metabolism on β-amyloid plaque formation and astrogliosis.

### *M. parviflora* hydroalcoholic extract ameliorates impairments in spatial memory in 5XFAD mice fed with a high fat diet

**Fig. 4:**
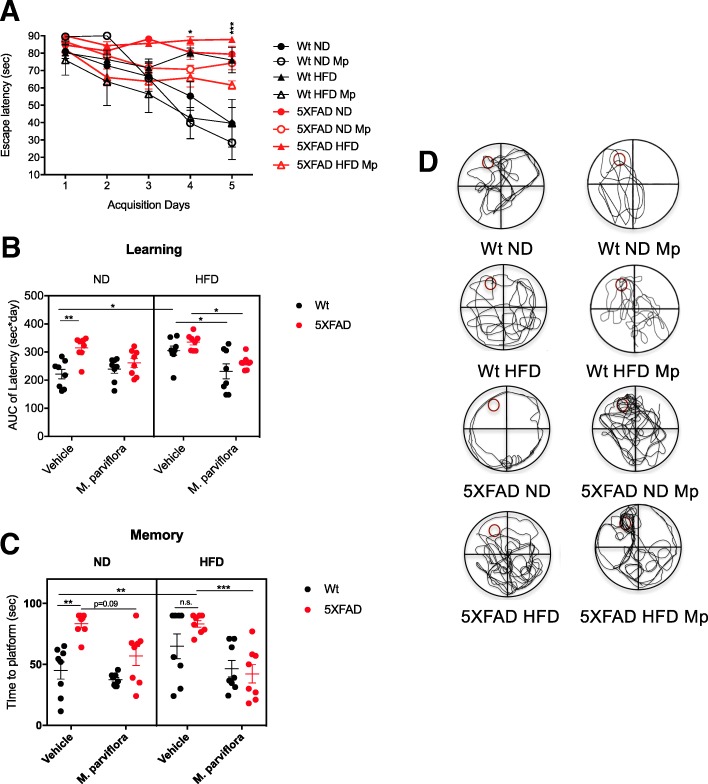
*Malva parviflora* hydroalcoholic extract protects 5XFAD transgenic mice from memory deficit. Morris Water Maze test was performed to evaluate spatial memory in 5XFAD mice treated with *Malva parviflora* hydroalcoholic extract (*M*. *parviflora*). **a** Time (sec) need to reach the hidden platform (escape latency) during the five acquisition days (test trial) of wild type (Wt) mice fed with normal diet (ND) (closed black cirle) or high-fat diet (HFD) (closed black triangle) and those that received intragastrically water or 50 mg/kg/day of the MpHE (Mp): Wt ND + Mp (open black circle) or Wt HFD + Mp (open black triangle), or transgenic 5XFAD mice fed with a normal diet (ND) (closed red circle) or a high-fat diet (HFD) (closed red triangle) that received intragastrically water or 50 mg/kg/day of the MpHE (Mp): 5XFAD ND + Mp (open red circle), or 5XFAD HFD + Mp (open red triangle). Data are shown as mean ± SEM, *n* = 8 animals per group. Statistical analysis was performed by two-way ANOVA followed by Sidak’s multiple comparisons test. This revealed a significant effect for time *F*(4,108) = 12.16, *p* < 0.0001, Genotype *F*(7,27) = 11.17, *p* < 0.0001 and for the interaction between time and genotype *F*(28,108) = 1.969, *p* = 0.007 (day 4 Wt ND *vs* 5XFAD HFD *p = 0.04; day 5 Wt ND *vs* 5XFAD HFD ***p < 0.0001). **b** Area under the curve (AUC) of the latencies for each group was calculated using the trapezoidal rule. Data are shown as mean ± SEM, *n* = 8 animals per group. Statistical analysis was performed by three-way ANOVA followed by Tukey’s multiple comparisons test. This analysis revealed a significant effect for the genotype *F*(1,56) = 14.95, *p < 0*.*001*; for the *M*. *parviflora* treatment *F*(1,56) = 15.69, *p < 0*.*001*; for the genotype and diet interaction *F*(1,56) = 1.401, *p = 0*.*24*; for the *M*. *parviflora* treatment and diet interaction *F*(1,56) = 5.734, *p = 0*.*02*; for the genotype and *M*. *parviflora* treatment interaction *F*(1,56) = 2.328, *p = 0*.*13*; for the genotype, *M*. *parviflora* treatment and diet interaction *F*(1,56) = 2.467, *p = 0*.*12*. **c** Time (seconds; sec) to platform for each group during the probe trial (day eight) in the absence of platform. Statistical analysis was performed by three-way ANOVA followed by Tukey’s multiple comparisons test. This analysis revealed a significant effect for the genotype *F*(1,56) = 15.46, *p < 0*.*001*; for the *M*. *parviflora* treatment *F*(1,56) = 25.97, *p < 0*.*001*; for diet *F*(1,56) = 0.5947, *p* = 0.44; for the genotype and diet interaction *F*(1,56) = 5.703, *p = 0*.*02*; for the *M*. *parviflora* treatment and diet interaction *F*(1,56) = 1.915, *p = 0*.*17*; for the genotype and *M*. *parviflora* treatment interaction *F*(1,56) = 5.131, *p = 0*.*03*; for the genotype, *M*. *parviflora* treatment and diet interaction *F*(1,56) = 0.04011, *p = 0*.*84*. **d** Representative swimming paths of mice during the probe trial on day eight are depicted. The hidden platform was located on the NW quadrant

Given that the MpHE attenuated the alterations in glucose metabolism and reduced Aβ deposition in 5XFAD mice fed with HFD, we tested whether this treatment could also impact spatial learning and memory. To this purpose, the Morris water maze test was performed. In the training trials, Wt mice fed with HFD took more time to find the platform than Wt mice fed with ND (*p =* 0.008) (Fig. 4a) and their path lengths were less directed to the platform (Fig. 4d). Notably, 9-month-old 5XFAD mice fed with ND present similar learning impairments when compared to Wt animals fed with HFD (Fig. 4a, b). 5XFAD animals fed with ND (*p = 0*.*003*) and HFD (*p = < 0*.*001*) showed higher escape latencies in the learning test (Fig. 4b) and longer times to the platform in the memory test (5XFAD ND *p = 0*.*002*; 5XFAD HFD *p < 0*.*002*) compared with Wt mice fed with ND (Fig. 4c). The HFD did not worsen memory loss in 9-month-old 5XFAD mice, since transgenic mice fed with ND or HFD had similar escape latencies to find the platform. Nonetheless, the MpHE improved spatial learning in 5XFAD mice fed with HFD because it reduced significantly the escape latency compared to untreated transgenic mice (*p = 0*.*05*) and the latency is even similar to that of the ND-fed Wt mice (Fig. 4b). In the probe trial assay, most of the 5XFAD mice fed with ND and treated with the MpHE, reached the quadrant where the platform was located in a shorter time than the untreated transgenic mice (Fig. 4c). The 5XFAD animals fed with HFD and treated with *M*. *parviflora* showed a considerable reduction of the escape latency (*p < 0*.*001*) compared with that of the 5XFAD HFD group (Fig. 4c). Accordingly, independent of the diet, transgenic mice administered with MpHE showed reduced path lengths and crossed over the location of the hidden platform more frequently compared with the untreated 5XFAD mice (Fig. 4d). The *M*. *parviflora* capacity to ameliorate the memory impairment of 5XFAD transgenic model mice was further evident in the *probe* trial assay, where the latencies of the *M*. *parviflora* treated transgenic mice were very similar to that of the ND-fed Wt mice (Fig. 4c). The MpHE had no effect on the cognitive capacities of Wt mice fed with a ND; however, Wt animals fed with HFD and treated with this extract showed a significant reduction (*p* = 0.04) in the escape latencies (Fig. 4b). Together these results indicate that the MpHE attenuates the learning and memory deficiencies resulting from Aβ deposition and altered glucose metabolism.

### *M. parviflora* hydroalcoholic extract regulates microglia activation in vitro

**Fig. 5:**
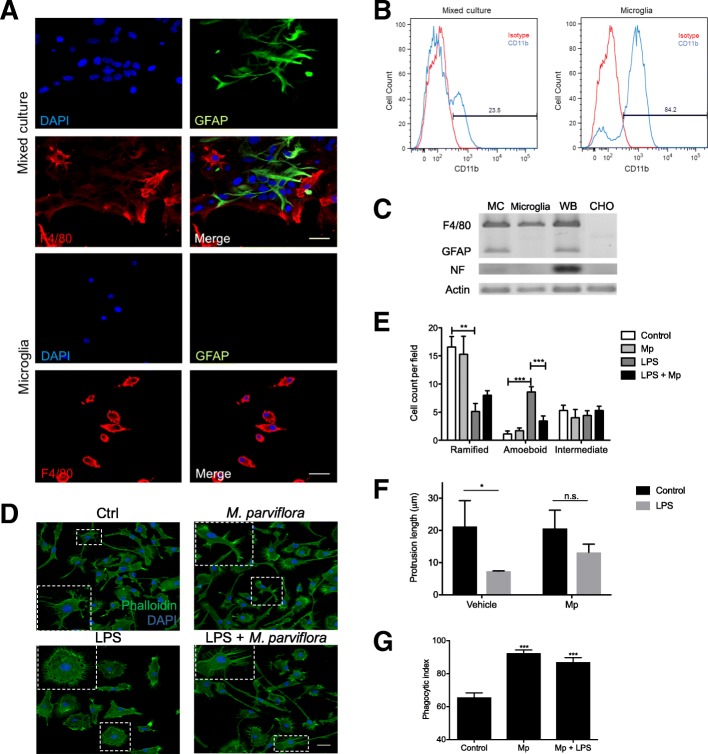
*Malva parviflora* hydroalcoholic extract regulates LPS-induced microglia activation. Microglia primary cultures were isolated from neonatal wild type animals as described in the “Methods” section. Microglia enrichment was determined as follows. **a** Confocal microscopy was used to examine GFAP (astrocytes) and F4/80 (microglia) expression in mixed cultures and after microglia purification (Microglia). Nuclei were visualized by DAPI staining (scale bar, 30 μm). **b** Microglia enrichment was determined by flow citometry using anti-CD11b antibodies. **c** The mRNA levels of different cell markers, microglia (F4/80); astrocytes (GFAP) and neurons (Neurofilament M; NF) were determined in the mixed cultures (MC), isolated microglia (microglia), whole brain (WB) and CHO cells by RT-PCR analysis as described in the “Methods” section. Actin levels were used as internal control. **d** Microglial cultures were exposed to PBS (Ctrl), LPS (100 ng/mL), MpHE (*M*. *parviflora*) (1 mg/mL) or LPS (100 ng/mL) and MpHE (1 mg/mL) (*LPS + M*. *parviflora*) for 24 h. Cells were fixed, stained with phalloidin and DAPI for F-actin (green) and nuclei (blue) detection, respectively. Microglia was imaged using the Olympus FluoView 1000 confocal multiphoton microscope (scale bar, 30 μm). The boxed areas were × 2 digitally magnified and shown as inset. **e** The microglia morphologies were classified as ramified, amoeboid and intermediate shapes in the different groups described in (D); MpHE (*Mp*). One hundred cells were measured for each experimental condition. Data (mean ± SEM) were analyzed by one-way ANOVA followed by Tukey’s post hoc test (^**^p < 0.01; ^***^p < 0.001). **f** Microglial cultures were exposed to PBS (Control) or LPS (100 ng/mL) in the presence or absence Vehicle) of MpHE (1 mg/mL) (*Mp*) for 24 h. The length of the protrusions (μm) of the microglia were determined using Neurite Tracer from ImageJ software (National Institutes of Health, Bethesda, MD). Data (mean ± SEM) were analyzed by two-way ANOVA followed by post hoc Bonferroni’s multiple comparisons test. This analysis revealed a significant effect for the LPS *F*(1,8) = 13.32, *p = 0*.*006*; not for the treatment with *M*. *parviflora F*(1,8) = 0.8054 *p = 0*.*40* or for the LPS and *M*. *parviflora* interaction *F*(1,8) = 1.245 *p = 0*.*30*. **g** Microglial cultures were exposed to PBS (Control), MpHE (1 mg/mL) (*Mp*) or LPS (100 ng/mL) and MpHE (1 mg/mL) (*LPS + Mp*) for 24 h. The phagocytic index was calculated by multiplying the percentage of microglia with internalized latex beads by the average number of internalized latex beads per each group. Data were collected from seven random fields per group and analyzed by one-way ANOVA followed by Tukey’s post hoc test (^***^p < 0.001 *vs* control)

Many studies indicate that the microglial cells, which are crucial in the CNS homeostasis by clearing misfolded proteins and controlling inflammation, have altered functions in the AD [32]. As first approach to determine whether MpHE could modulate microglia activation and thus attenuate memory loss in the 5XFAD mice, microglia primary cultures were prepared from brain of neonatal mice. Microglia isolation was determined by confocal microscopy (Fig. 5a), flow cytometry (Fig. 5b), and RT-PCR (Fig. 5c) assays using different cell markers, including F4/80 and CD11b (microglia), GFAP (astrocytes), and Neurofilament M (neurons). Purified microglia was evidenced by the expression of F4/80 and CD11b (Fig. 5a–c). Using these cultures, we evaluated the effect of the MpHE on LPS-induced microglia activation. Phalloidin staining revealed that LPS treatment induced microglia morphological changes by increasing the amoeboid “activated” morphology (*F*(3,24) = 20.49, *p < 0*.*001*) and reducing the ramified morphology (*F*(3,24) = 7.526, *p < 0*.*01*) as compared with the control cells (Fig. 5d, e). In contrast, when MpHE was added to the microglial cultures, the LPS-induced amoeboid morphological changes were significantly reduced (*F*(3,24) = 20.49, *p < 0*.*001*) (Fig. 5d, e). In the same way, LPS treatment reduced the protrusion length of the microglia processes (*p = 0*.*0195*) compared with control microglial cells that had fine processes towards the trailing end (Fig. 5f). The MpHE treatment prevented the reduction in the protrusion length resulting from LPS exposure (Fig. 5f). Even more, LPS-treated microglia exposed to MpHE showed enhanced phagocytic activity when compared to untreated control cells (*F*(2,15) = 30.67, *p < 0*.*001*) (Fig. 5g). Interestingly, *M*. *parviflora* treated microglia showed increased phagocytic activity similar to that observed in microglial cells treated with LPS and *M*. *parviflora* (*F*(2,15) = 30.67, *p < 0*.*001*) (Fig. 5g). Together, these results indicate that the MpHE modulates microglia activation in response to inflammatory stimuli while promoting its phagocytic activity.

### *M. parviflora* hydroalcoholic extract stimulates the microglia phagocytic activity from 5XFAD mice

**Fig. 6:**
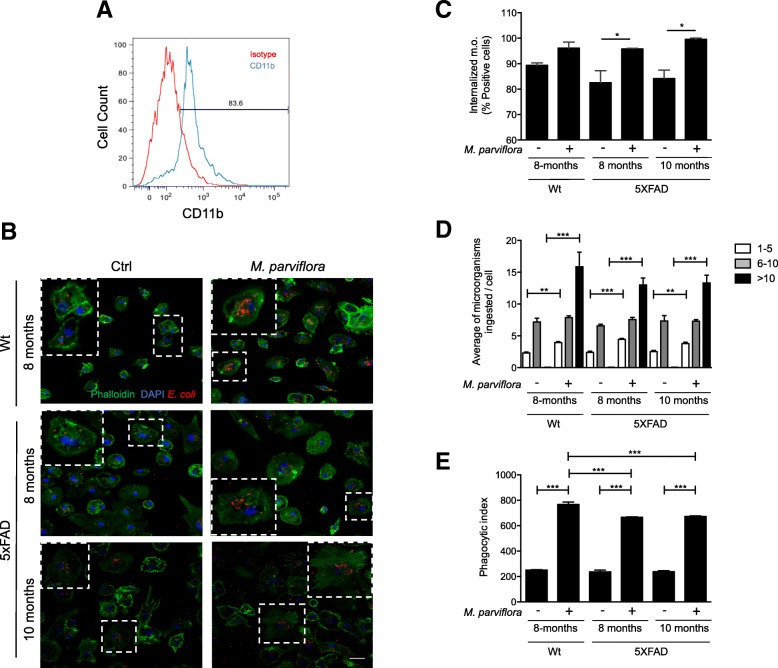
Ex vivo treatment of microglial cells from 5XFAD mice with *Malva parviflora* hydroalcoholic extract increases the microglia phagocytic activity. **a** Microglia was purified from adult (wild type and 5XFAD) mouse brains as described in the “Methods” section. Microglia enrichment was determined by flow citometry using anti-CD11b antibodies. A representative histogram depicts isotype (red) and CD11b (blue) labeled cells from 5XFAD transgenic brain. **b** Primary microglial cells were purified from 8- and 10-months-old Wt and 5XFAD mice as described in the “Methods” section and left untreated (Ctrl) or treated with the *M*. *parviflora* extract (1 mg/mL) for 24 h and then exposed to fluorescent *E*. *coli* (red) for 4 h. Cells were fixed and stained with phalloidin and DAPI for F-actin (green) and nuclei (blue) detection, respectively. Microglia was imaged using the Olympus FluoView 1000 confocal multiphoton microscope (Scale bar, 30 μm). Insets show a × 2 digital magnification from boxed areas. **c** Graph depicts the percentage of cells with internalized *E*. *coli* (m.o.) that were left untreated (−) or treated (+) with the *M*. *parviflora* extract (1 mg/mL) for 24 h and then exposed to fluorescent *E*. *coli* (red) for 4 h. Data (mean ± SEM) were analyzed by one-way ANOVA followed by Tukey’s post hoc test (^*^p < 0.05). **d** Average of microorganisms (*E*. *coli*) internalized by microglia from the indicated mouse strain and age untreated (−) or treated (+) with the *M*. *parviflora* extract (intervals were from 1 to 5, from 6 to 10 and greater than 10 microorganisms/cell). Data (mean ± SEM) were analyzed by paired *t* test (^**^p < 0.01; ^***^p < 0.001). **e** Phagocytic index was calculated by multiplying the percentage of microglia with internalized bacteria by the average number of internalized *E*. *coli* bacteria per each group. Data were collected from seven random fields per group and analyzed by one-way ANOVA followed by Tukey’s post hoc test (^***^p < 0.001)

Taking into consideration that the inflammatory environment induced by Aβ-accumulation skews microglia to an inflammatory phenotype impairing its phagocytic capacity [33, 34] thus leading to the AD progression and that MpHE induced the phagocytic activity of neonatal microglia in primary cultures (Fig. 5), we evaluated whether MpHE could restore the phagocytic activity of microglia from adult 5XFAD mice. For this purpose, pure primary microglial cultures from 8- and 10-month-old Wt or 5XFAD mice were isolated (Fig. 6a) and exposed to fluorescent *E*. *coli* in the present or absence of the MpHE. Interestingly, the percentage of microglia with internalized bacteria was slightly reduced in the cultures of microglia isolated from brains with Aβ deposition (5XFAD mice, 8 and 10 months old) when compared to cultures of microglia isolated from healthy brains (Wt mice) (Fig. 6b, c), thus confirming that an inflammatory environment impairs microglia phagocytic activity [9]. However, exposure to the MpHE increased the number of cells with internalized bacteria in 5XFAD microglial cultures from 8- (*F*(5,12) = 7.351, *p < 0*.*05*) and 10-month-old mice (*F*(5,12) = 7.351, *p < 0*.*05*) (Fig. 6b, c). Furthermore, the MpHE significantly increased the number of cells containing more than 10 internalized *E*. *coli* in both, Wt and 5XFAD microglia (F(5,294) = 86.56, Wt 1-5, *p < 0*.*01*; Wt 6-10, *p < 0*.*001*; 5XFAD 8-month-old 1–5, *p < 0*.*001*; 5XFAD 8-month-old 6–10, *p < 0*.*001*; 5XFAD 10-month-old 1–5, *p < 0*.*01*; 5XFAD 10-month-old 6–10, *p < 0*.*001*) (Fig. 6d). Additionally, the phagocytic index was increased for the presence of MpHE in all the cases (*F*(5,12) = 620, *p < 0*.*001* for all the cases) (Fig. 6e). Thus, our results indicate that the negative effect of the MpHE on Aβ plaques formation may result from improved microglia phagocytic activity and Aβ peptide clearance.

### *M. parviflora* hydroalcoholic extract reduces the M1 phenotype in the microglial cells from 5XFAD mice

**Fig. 7:**
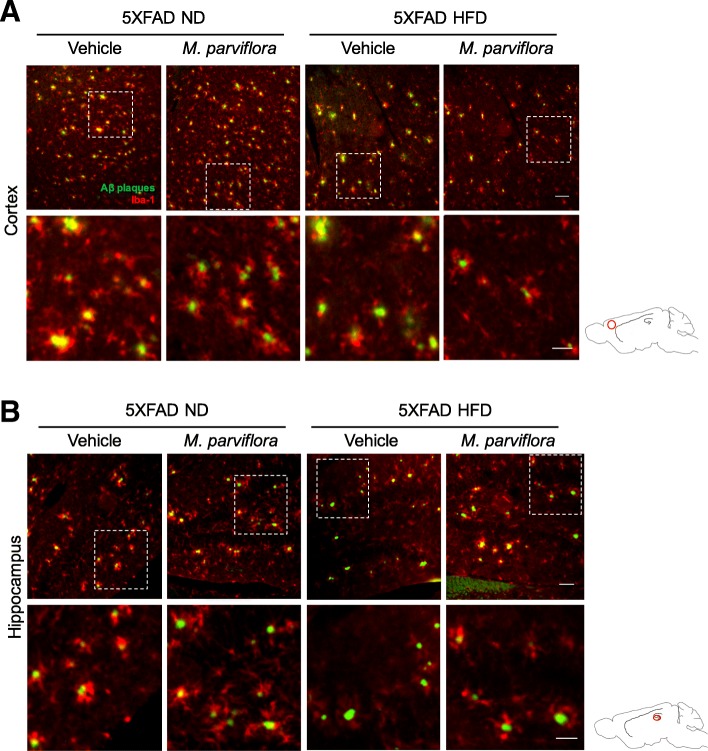
*Malva parviflora* hydroalcoholic extract increases microglia accumulation around the Aβ plaques in the cortex and hippocampus of 5XFAD mice. Amyloid plaques were labeled with thioflavin S (green) and microglia with anti-Iba-1 antibodies (red) on cortical **a** or hippocampal sections (dentate gyrus) **b** from 5XFAD mice fed with either normal diet (ND) or high-fat diet (HFD) non-treated (Vehicle) or treated with *M*. *parviflora* extract (*M*. *parviflora*) for 8 months. Representative micrographs are depicted. Images were captured using the Zeiss Axioskop Observer Z1 inverted fluorescence microscope (× 10, scale bar 100 μm). Lower row represents a × 3 digital magnification from boxed areas (scale bar 30 μm). Sketch indicates the analyzed area within the cortex and the hippocampus, respectively

**Fig. 8:**
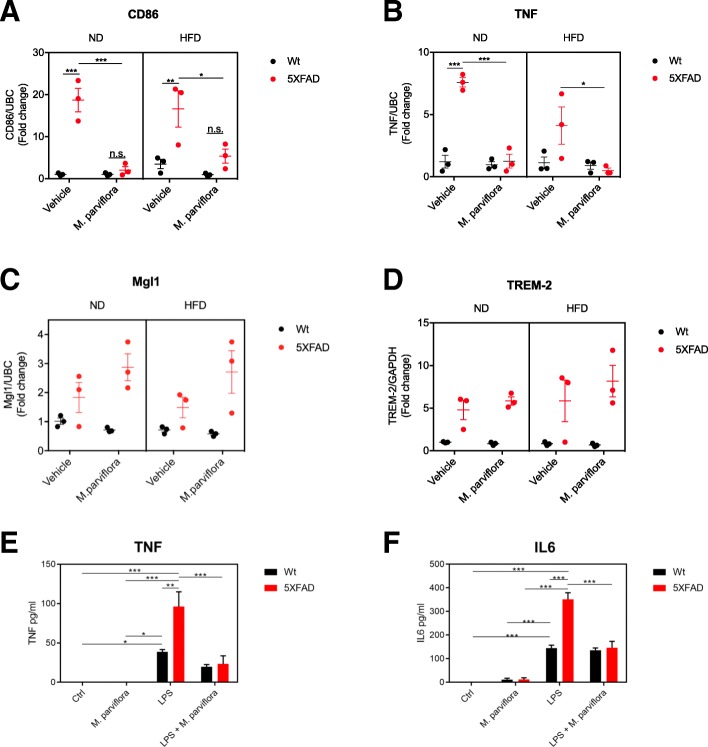
*Malva parviflora* hydroalcoholic extract attenuates microglia pro-inflammatory M1 phenotype in the cortex of 5XFAD mice. Total RNA was isolated from the cortex of Wt or 5XFAD mice fed with either normal diet (ND) or high-fat diet (HFD) non-treated (Vehicle) or treated with MpHE (*M*. *parviflora*) for 8 months. **a** The transcript levels of CD86 (marker of M1 state) were determined by RT-qPCR as described in the “Methods” section. Data are shown as mean ± SEM, *n* = 3 animals per group. Statistical analysis was performed by three-way ANOVA followed by Tukey’s multiple comparisons test. This analysis revealed a significant effect for the genotype *F*(1,16) = 42.45, *p < 0*.*001*; for the diet *F*(1,16) = 0.4022, *p = 0*.*53*; for the *M*. *parviflora* treatment *F*(1,16) = 29.79, *p < 0*.*001*; for the genotype and diet interaction *F*(1,16) = 0.04041, *p = 0*.*84*; for the *M*. *parviflora* treatment and diet interaction *F*(1,16) = 0.2594 *p = 0*.*62*; for the genotype and *M*. *parviflora* treatment interaction *F*(1,16) = 20.67 *p < 0*.*001*; for the genotype, *M*. *parviflora* treatment and diet interaction *F*(1,16) = 2.037, *p = 0*.*17*. **b** TNF (marker of M1 state) mRNA levels. Data are shown as mean ± SEM, *n* = 3 animals per group. Statistical analysis was performed by three-way ANOVA followed by Tukey’s multiple comparisons test. This analysis revealed a significant effect for the genotype *F*(1,16) = 25.65 *p < 0*.*001*; for the diet *F*(1,16) = 5.758, *p = 0*.*03*; for the *M*. *parviflora* treatment *F*(1,16) = 32.4, *p < 0*.*001*; for the genotype and diet interaction *F*(1,16) = 4.955, *p = 0*.*04*; for the *M*. *parviflora* treatment and diet interaction *F*(1,16) = 2.259 *p = 0*.*15*; for the genotype and *M*. *parviflora* treatment interaction *F*(1,16) = 26.77 *p < 0*.*001*; for the genotype, *M*. *parviflora* treatment and diet interaction *F*(1,16) = 2.189, *p = 0*.*16*. **c** Mgl1 (marker of M2 state) mRNA levels. Data are shown as mean ± SEM, n = 3 animals per group. Statistical analysis was performed by three-way ANOVA followed by Tukey’s multiple comparisons test, and **d** TREM-2 mRNA levels. Data are shown as mean ± SEM, n = 3 animals per group. Statistical analysis was performed by three-way ANOVA followed by Tukey’s multiple comparisons test. Microglia from 8-month-old Wt or 5XFAD mice were unstimulated or stimulated with LPS (100 ng/mL) in the presence or absence of MpHE (*M*. *parviflora*; 1 mg/mL) for 24 h. Control cells were treated with PBS (Ctrl) or MpHE alone (*M*. *parviflora*). Supernatants were used to determine TNF and IL6 levels by ELISA as described in the “Methods” section. **e** TNF levels. Data are shown as mean ± SEM, n = 3 animals per group. Statistical analysis was performed by three-way ANOVA followed by Tukey’s multiple comparisons test. This analysis revealed a significant effect for the genotype *F*(1,16) = 7.878, *p = 0*.*0127*; for the LPS treatment *F*(1,16) = 17.74, *p = 0*.*0007*; for the *M*. *parviflora* treatment *F*(1,16) = 66.30, *p < 0*.*0001*; for the genotype and LPS treatment interaction *F*(1,16) = 6.105, *p = 0*.*0251*. **f** IL6 levels. Data are shown as mean ± SEM, n = 3 animals per group. Statistical analysis was performed by three-way ANOVA followed by Tukey’s multiple comparisons test. This analysis revealed a significant effect for the genotype *F*(1,16) = 25.76, *p = 0*.*0001*; for the LPS treatment *F*(1,16) = 19.86, *p = 0*.*0004*; for the *M*. *parviflora* treatment *F*(1,16) = 309.3, *p < 0*.*0001*; for the genotype and LPS treatment interaction *F*(1,16) = 20.71, *p = 0*.*0003*

Previous reports showed that the microglial cells in the presence of Aβ plaques exhibit morphological and molecular changes, their soma and the thickness processes are increased [35] as well as the expression of inflammatory mediators a characteristic of an activated M1 phenotype that result in neurotoxicity [36]. Hence, we evaluated if the MpHE could also modulate these alterations. We used Iba-1 as specific marker to analyze the microglia morphological changes in cortex and hippocampus of 5XFAD mice untreated or treated (8 months) with the MpHE. In contrast to the activated microglia (amoeboid) observed in untreated 5XFAD mice, the presence of ramified microglia with long and thin processes within the cortex and hippocampus of 5XFAD mice exposed to MpHE was evident (Fig. 7a, b). Importantly, an increased microglia accumulation around the Aβ plaques was observed in the cortex and hippocampus of 5XFAD mice treated with the MpHE as determined by the Manders’s coefficient (hippocampus 5XFAD mice 0.774 ± 0.105 *vs* 5XFAD mice treated with *M*. *parviflora* 0.875 ± 0.084; cortex 5XFAD mice 0.674 ± 0.076 *vs* 5XFAD mice treated with *M*. *parviflora* 0.894 ± 0.035) (Fig. 7a, b). This correlated with reduced thioflavin-S staining intensity (Fig. 3a), suggesting improved Aβ plaques degradation. Moreover, the increased mRNA levels of pro-inflammatory markers as CD86 (Wt ND *vs* 5XFAD ND *p < 0*.*001*; Wt HFD *vs* 5XFAD HFD *p = 0*.*0045*), which is expressed by the microglial cells in the brain [37], and TNF [38] (Wt ND *vs* 5XFAD ND, *p < 0*.*001*) observed in the cortex of 5XFAD mice independent of the diet were reduced in the cortex from 5XFAD mice treated with MpHE (CD86: 5XFAD ND *vs* 5XFAD ND *M*. *parviflora* (*p < 0*.*001*) and 5XFAD HFD *vs* 5XFAD HFD *M*. *parviflora* (*p = 0*.*0167*) (Fig. 8a); TNF: 5XFAD ND *vs* 5XFAD ND treated with *M*. *parviflora*, *p < 0*.*001*; 5XFAD HFD *vs* 5XFAD HFD treated with *M*. *parviflora*, *p = 0*.*02*) (Fig. 8b). In contrast, the levels of Mgl1 (macrophage galactose-type lectin-1), an anti-inflammatory microglia marker [39], showed a mild increase in the presence of the extract in the 5XFAD fed with a ND or HFD (Fig. 8c). Also, we analyzed the mRNA levels of TREM-2 (triggering receptor expressed on myeloid cells-2), a phagocytosis specific marker in microglial cells [40]. We found that TREM-2 levels were increased in the cortex from 5XFAD mice and the *M*. *parviflora* extract treatment slightly further increased TREM-2 levels in lean and obese mice (Fig. 8d).

To further corroborate the anti-inflammatory effect of MpHE, primary microglial cultures from 5XFAD mice (8 months) were treated con LPS (100 ng/mL) in the absence or presence of MpHE (1 mg/mL) for 24 h. Interestingly, although LPS promoted higher production of both TNF and IL-6 in microglia from the 5XFAD mice than that from wild type mice, the increased levels of TNF and IL-6 released to the supernatant in response to LPS (TNF: Wt Ctrl *vs* Wt LPS *p = 0*.*0434*; 5XFAD Ctrl *vs* 5XFAD LPS *p < 0*.*0001*; Wt LPS *vs* 5XFAD LPS *p = 0*.*0015*; and IL-6: Wt Ctrl *vs* Wt LPS *p = 0*.*0001*; 5XFAD Ctrl *vs* 5XFAD LPS *p < 0*.*0001*; Wt LPS *vs* 5XFAD LPS *p < 0*.*0001*) was significantly reduced in response to MpHE (TNF: 5XFAD LPS *vs* 5XFAD LPS treated with *M*. *parviflora*, *p = 0*.*0001*; and IL-6: 5XFAD LPS *vs* 5XFAD LPS treated with *M*. *parviflora*, *p < 0*.*0001*) (Fig. 8e, f). According with this, the in vivo treatment with the MpHE also reduced the inflammatory process in the adipose tissue of wild type and 5XFAD mice fed with the HFD, since there was a clear reduction in the infiltration of immune cells into the adipose tissue of mice fed with HFD and treated with the MpHE compared with vehicle-treated animals (Additional file 2: Figure S2). Together these results indicate that (1) by regulating microglia phagocytic activity, (2) by preventing the pro-inflammatory M1 phenotype, *M*. *parviflora* reduces β-amyloid plaque load and improves learning and memory in lean and obese mice and (3) *M*. *parviflora* attenuates the inflammatory process in the adipose tissue of obese mice.

### Scopoletin and oleanolic acid are present in the *M. parviflora* hydroalcoholic extract

**Fig. 9:**
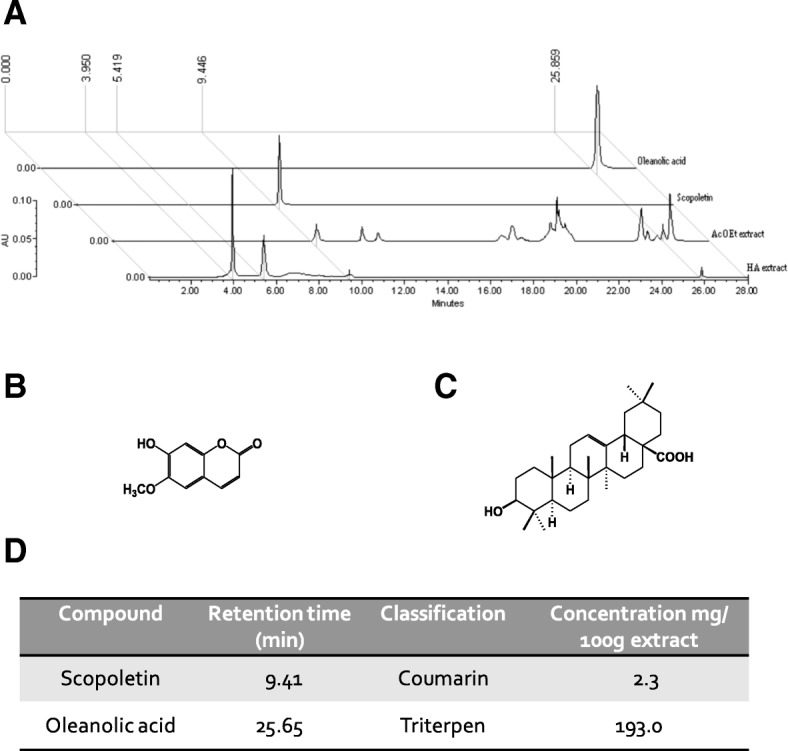
The *Malva parviflora* hydroalcoholic extract contains scopoletin and oleanolic acid. **a** HPLC chromatograms comparing the hydroalcoholic (HA) and ethyl acetate (AcOEt) fractions from MpHE with reference standards of scopoletin and oleanolic acid. All samples were monitored at 345 nm. **b** Chemical structure of scopoletin. **c** Chemical structure of oleanolic acid. **d** Principal compounds present in the hydroalcoholic extract of *M*. *parviflora*

Given that *M*. *parviflora* reduces neuroinflammation and regulates microglia activation in the 5XFAD mice, the fractionation and characterization of the MpHE was performed to ascertain the bioactive components. MpHE was subjected to a series of solvent extraction and the water and ethyl acetate fractions were subjected to HPLC analysis to identify compounds with potential anti-inflammatory or anti-oxidant activities. Scopoletin was identified in the ethyl acetate (AcOet) fraction (Fig. 9a). This coumarin displayed the same retention time (9.4 min) and UV spectra (*λ* max = 205, 228, 294, 348 *n*m) than the commercial reference (Fig. 9b). Oleanolic acid was also identified in the same fraction (Fig. 9a), showing the same retention time (25.85 min) and UV (*λ* max = 192 nm) spectra than the commercial reference (SIGMA, 95% purity) (Fig. 9c). Additionally, the presence of two peaks in the early retention times, displayed on the UV spectra 3.95 min (*λ* = 204, 276, 369 nm) and 5.4 min (*λ* = 200, 220, 279), is related to flavonoid compounds. Quantification of scopoletin was established with calibration curve (linear regression displayed the equation *Y* = 84,221*X* − 188,219 *R*^*2*^ = 0.9995) of this coumarin. Based on the obtained equation *Y* = 84,221*X* − 188,219, it is possible to establish a 1.22 mg of scopoletin content per gram of AcOet fraction and 101.9 mg of oleanolic acid content per gram of AcOet fraction. Considering the yield of the HE (1 g of HE produces 0.019 g of AcOet fraction), the scopoletin concentration corresponds to 23 μg/g of HE and 1.93 mg/g of HE for the oleanolic acid (Fig. 9d). According to previous data indicating that oleanolic acid and scopoletin have anti-inflammatory activity [41, 42], both compounds inhibited LPS-induced NF-κB activity in mouse RAW-Blue macrophages (Additional file 3: Figure S3).

These data indicate that the anti-neuroinflammatory effect of the MpHE, observed in this study, might result from the presence of oleanolic acid and scopoletin.

### *M*. *parviflora* hydroalcoholic extract regulates the phagocytic capacity of microglial cells via the PPARγ-CD36 pathway

**Fig. 10:**
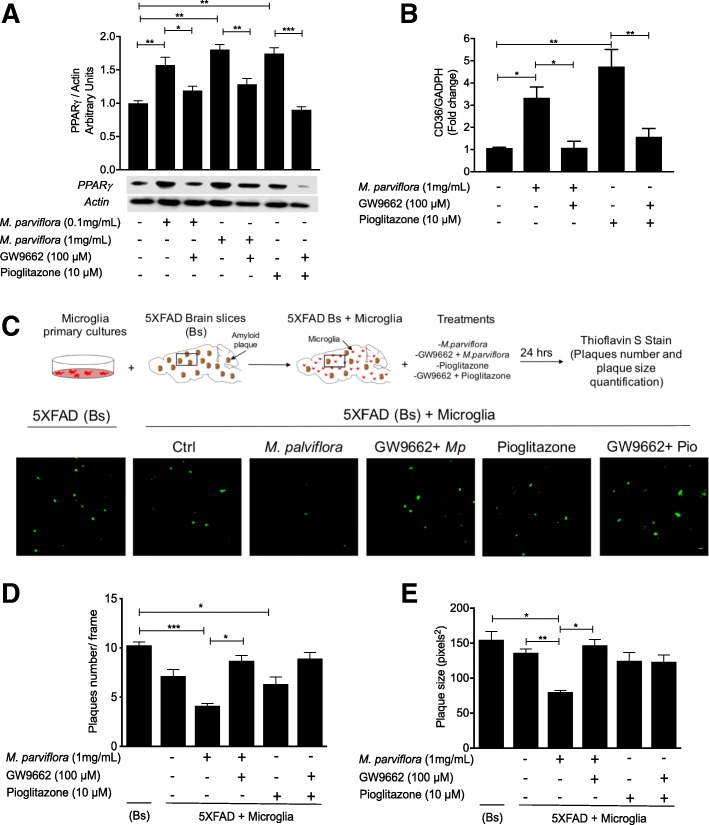
*Malva parviflora* hydroalcoholic extract regulates the phagocytic capacity of microglial cells via PPARγ-CD36 mediated mechanism. Microglial primary cultures were left untreated or pre-treated with GW9662, a specific PPAR-γ inhibitor for 1 h, following by incubation with MpHE or the PPAR-γ agonist, pioglitazone, at the indicated concentration for 24 h. **a** The PPAR-γ levels from the cell extracts of microglial primary cultures were determined by immunoblot using specific antibodies and the actin levels were used as internal control. Normalized densitometry values of PPAR-γ (PPAR-γ/actin) present in the extracts of microglial primary cultures in the presence of MpHE (Mp) or pioglitazone and GW9662. Data are shown as mean ± SEM and were analyzed by one-way ANOVA. Ctrl *vs* Mp 0.1 mg/mL (**p = 0.009); Ctrl *vs* Mp 1 mg/mL (**p = 0.004); Ctrl *vs* pioglitazone (**p = 0.001); Mp 0.1 mg/mL *vs* GW9662 + Mp 0.1 mg/mL (*p = 0.04); Mp 1 mg/mL *vs* GW9662 + Mp 1 mg/mL (**p = 0.003); Pioglitazone *vs* GW9662 + Pioglitazone (***p < 0.001). **b** CD36 mRNA levels were determined by qPCR using total RNA from the microglial primary cultures treated as described above. Data were analyzed by one-way ANOVA. Ctrl *vs* Mp (*p = 0.04); Ctrl *vs* pioglitazone (**p = 0.002); Mp *vs* GW9662 + Mp (*p = 0.04); pioglitazone *vs* GW9662 + pioglitazone (**p = 0.005). **c** Representative micrographs from cortex of adult 5XFAD mice alone (Bs) or in the presence of microglial primary cultures (5XFAD Bs + microglia) untreated (−) or pre-treated with *M*. *parviflora* or pioglitazone and GW9662. The slices were stained with thioflavin S and analyzed by confocal microscopy. **d** The plaques number were quantified. Data were analyzed by one-way ANOVA. Bs *vs M*. *parviflora* (***p < 0.001); Bs *vs* pioglitazone (*p = 0.03); *M*. *parviflora vs* GW9662 + *M*. *parviflora* (*p = 0.04). **e** The plaques size was quantified. Data were analyzed by one-way ANOVA. Bs *vs M*. *parviflora* (*p = 0.03); Ctrl *vs M*. *parviflora* (**p = 0.007); *M*. *parviflora vs* GW9662 + *M*. *parviflora* (*p = 0.02)

As shown, chemical characterization of MpHE determined the presence of oleanolic acid and scopoletin. Oleanolic acid is a natural agonist of the nuclear receptor peroxisome proliferator-activated receptor gamma (PPAR-γ) [43, 44]. Interestingly, PPAR-γ, through the induction of CD36 expression, mediates microglial amyloid β plaques phagocytosis in the APP/PS1 mice [16]. Since MpHE stimulates the microglia phagocytosis activity from 5XFAD mice, we tested whether the improved microglial phagocytic activity resulting from MpHE involved PPAR-γ activation. In accordance with this idea, MpHE increased the levels of PPAR-γ in a concentration-dependent manner (Ctrl *vs M*. *parviflora* 0.1 mg/mL; *p = 0*.*009*; Ctrl *vs M*. *parviflora* 1 mg/mL, *p = 0*.*004*) (Fig. 10a) and to a similar extent than the PPAR-γ agonist pioglitazone (*p = 0*.*001*) (Fig. 10a). In accordance with the fact that PPAR-γ induces its own expression through a positive feedback loop [45], the PPAR-γ-specific inhibitor GW9662 (100 μM) prevented the increase in PPAR-γ levels resulting from the MpHE treatment (*M*. *parviflora* 0.1 mg/mL *vs* GW9662 + *M*. *parviflora* 0.1 mg/mL (*p =* 0.04); *M*. *parviflora* 1 mg/mL *vs* GW9662 + *M*. *parviflora* 1 mg/mL (*p =* 0.003), as well as that induced by pioglitazone (*p <* 0.001) (Fig. 10a). GW9662 acts as a potent antagonist of PPAR-γ by covalently modifying a cysteine residue in the ligand binding site of PPAR-γ [46]. These results show that MpHE regulates PPAR-γ activity.

Since CD36 is a scavenger receptor involved in Aβ phagocytosis [16], whose expression is controlled by PPAR-γ [47], we tested the idea that the MpHE via PPAR-γ regulates CD36 expression. Microglia primary cultures were left untreated or pre-treated with GW9662 for 1 h, then incubated with MpHE (1 mg/mL) for 24 h and CD36 mRNA levels were measured. CD36 mRNA levels were upregulated in response to MpHE (*p =* 0.04) but prevented by GW9662 (*p = 0*.*04*) (Fig. 10b). The CD36 expression levels induced by the MpHE were similar to those resulting from exposing microglial cell to pioglitazone (*p =* 0.002) (Fig. 10b).

**Fig. 11:**
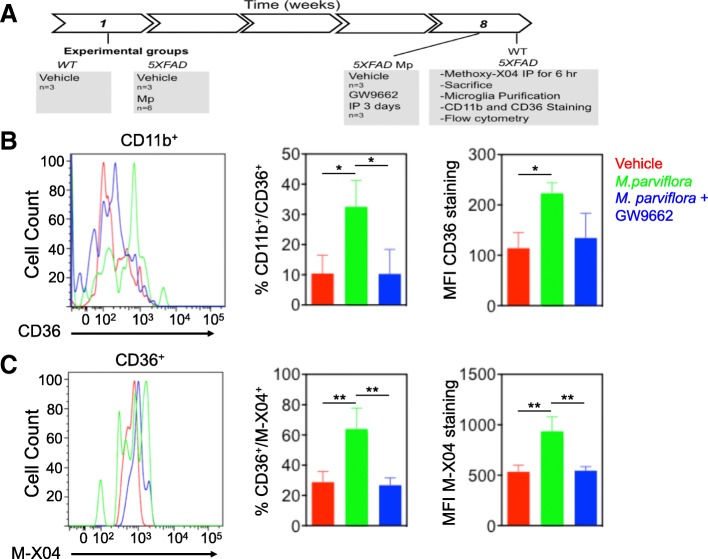
*Malva parviflora* hydroalcoholic extract regulates the phagocytic capacity of microglial cells via PPARγ-CD36 mediated mechanism in the 5xFAD transgenic mice. **a** Graphical time line of the study design and experimental procedures. The 5XFAD transgenic mice received 50 mg/kg/day of the MpHE (Mp) or water (Vehicle) intragastrically during 2 months. After, the mice were intraperitoneally (IP) injected with GW9662 (5 mg/kg), a specific PPAR-γ inhibitor, or vehicle (5% DMSO/95%PBS) for the last 3 days before sacrifice. For the in vivo amyloid β phagocytosis assay, mice were intraperitoneally injected 6 h before sacrifice with methoxy-X04 (10 mg/kg). The presence of Aβ peptides in the microglial cells were analyzed by flow cytometry. **b** CD36 expression in microglia (CD11b^+^) from 5XFAD transgenic mice untreated (Vehicle) or treated with MpHE alone (*M. parviflora*) or with MpHE and GW9662 (*M. parviflora*+GW9662) determined by flow cytometry. Left panel: A representative histogram depicts CD36 expression in the CD11b^+^ cells from 5XFAD transgenic brain**.** Middle panel: % of the CD11b^+^/CD36^+^ cells. Data (mean ± SD) were analyzed by one-way ANOVA followed by Tukey’s post hoc test. Percentage CD11b+/CD36+ 5XFAD vehicle *vs* 5XFAD Mp (*p = 0.0298); 5XFAD Mp *vs* 5XFAD Mp + GW9662 (*p = 0.0291). Right panel: CD36 expression levels. MFI CD11b^+^/CD36^+^ 5XFAD Vehicle *vs* 5XFAD Mp (*p = 0.0228). **c** Left panel: cytometry analysis of Aβ peptides phagocytized by microglia (CD11b+/CD36+) from adult 5XFAD mice untreated (Vehicle) alone (*M. parviflora*) or with MpHE and GW9662 (*M. parviflora*+GW9662) that received methoxy-X04 (M-X04). Left panel: representative histogram is shown. Middle panel: percent of the CD36^+^/M-X04^+^ cells data (mean ± SD) were analyzed by one-way ANOVA followed by Tukey’s post hoc test. Percentage CD36^+^/Methoxy-X04^+^ 5XFAD vehicle *vs* 5XFAD Mp (**p = 0.0089); 5XFAD Mp *vs* 5XFAD Mp + GW9662 (**p = 0.0069). Right panel: phagocyted M-XO4 levels. MFI CD36^+^/Methoxy-X04 5XFAD Vehicle *vs* 5XFAD Mp (**p = 0.0047); 5XFAD Mp *vs*. 5XFAD Mp + GW9662 (**p = 0.0054)

**Fig. 12:**
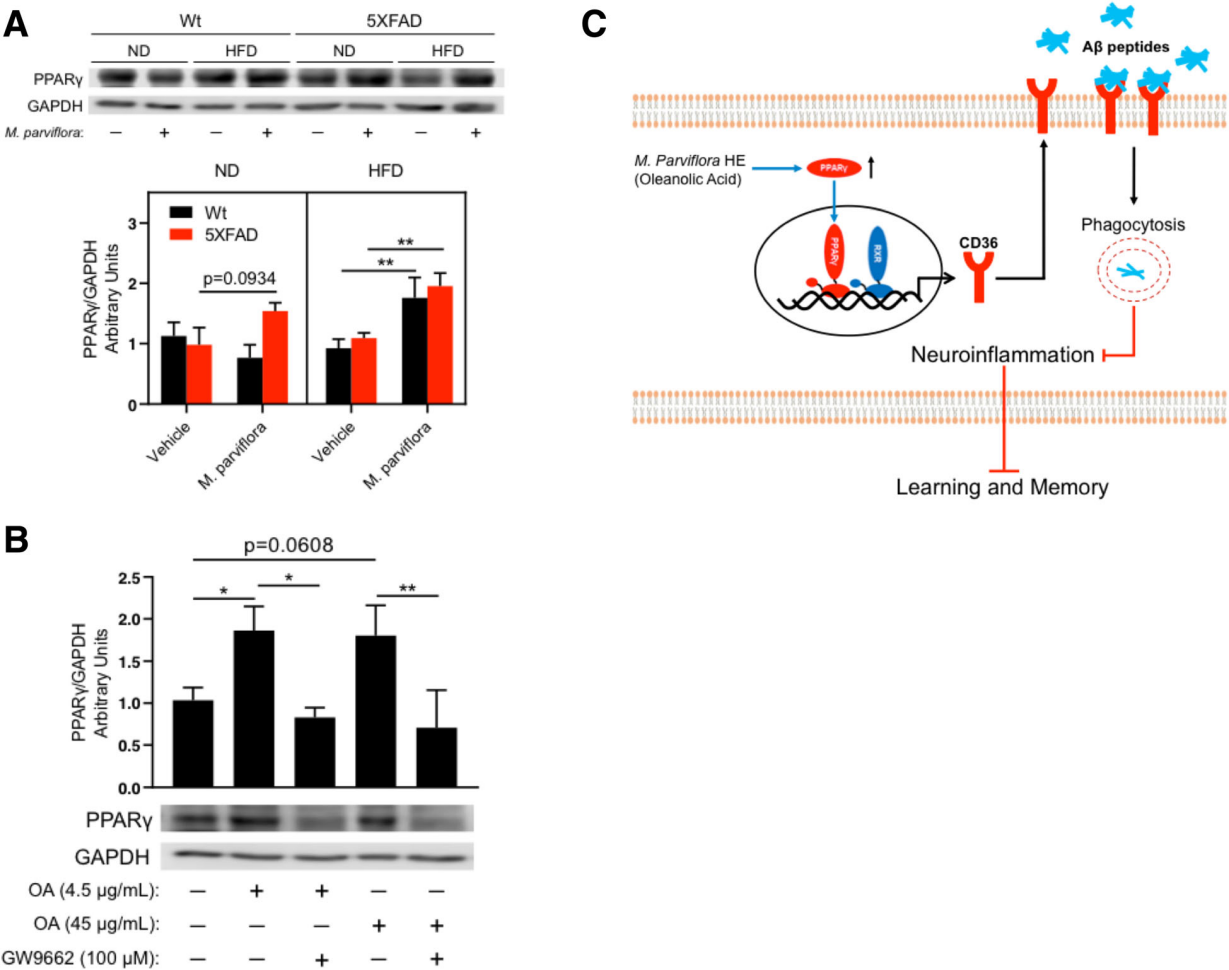
*Malva parviflora* hydroalcoholic extract regulates PPARγ levels in the 5XFAD transgenic mice. The 5XFAD and Wt mice were fed with either normal diet (ND) or high-fat diet (HFD) non-treated (Vehicle) or treated with *M*. *parviflora* hydroalcoholic extract (*M*. *parviflora*). The PPAR-γ levels from the frontal cortex cell extracts were determined by immunoblot using specific antibodies and the GAPDH levels were used as internal control. **a** Normalized densitometry values of PPAR-γ (PPAR-γ/GAPDH) present in the frontal cortex cell extracts of 5XFAD and Wt mice. Data (mean ± SD) were analyzed by three-way ANOVA followed by Tukey’s multiple comparisons test. This analysis revealed a significant effect for the genotype *F*(1,16) = 7.529, *p = 0*.*0144*; for the diet *F*(1,16) = 13.37, *p = 0-0021*; for the *M*. *parviflora* treatment *F*(1,16) = 28.11, *p < 0*.*0001*; for the genotype and diet interaction *F*(1,16) = 0.5415, *p = 0*.*4725*; for the *M*. *parviflora* treatment and diet interaction *F*(1,16) = 17.37, *p = 0*.*0007*; for the genotype and *M*. *parviflora* treatment interaction *F*(1,16) = 6.946, *p < 0*.*0180*; for the genotype, *M*. *parviflora* treatment and diet interaction *F*(1,16) = 6.111, p = 0.0250. **b** Microglial primary cultures were left untreated or pre-treated with GW9662, a specific PPAR-γ inhibitor for 1 h, following by incubation with oleanolic acid (OA), at the indicated concentration for 24 h. The PPAR-γ levels from the cell extracts of microglial primary cultures were determined by immunoblot using specific antibodies and the GAPDH levels were used as internal control. Normalized densitometry values of PPAR- γ (PPAR- γ/GAPDH) present in the extracts of microglial primary cultures in the presence of oleanolic acid. Data (mean ± SD) were analyzed by one-way ANOVA followed by Tukey’s post hoc test. Ctrl *vs* 4.5 μg/mL oleanolic acid (*p = 0.0414); 4.5 μg/mL oleanolic acid *vs* GW9662 + oleanolic acid 4.5 μg/mL (*p = 0.0116); 45 μg/mL oleanolic acid *vs* GW9662 + 45 μg/mL oleanolic acid (**p = 0.0079); **c** Model of the mechanism by which *M*. *parviflora* hydroalcoholic extract (HE) diminishes neuroinflammation. Chronic inflammation compromises microglia clearance functions by reducing the expression of the scavenger receptor CD36. The peroxisome proliferator-activated receptor (PPAR-γ) suppress inflammatory gene expression and promotes phagocytosis by regulating CD36 expression a scavenger receptor involved in microglia-dependent amyloid plaque destruction. According with this, our results indicate that a component present in the *M*. *parviflora* extract, probably oleanolic acid, based on previous studies [43] and our data, induces PPAR-γ activation that results in the upregulation of the scavenger receptor CD36 expression, thus leading to microglia-enhanced phagocytic, amyloid plaque clearance activity, diminished neuroinflammation, and improved learning and memory

Next, we evaluated whether PPR-γ activity was required for the enhanced microglia phagocytic activity mediated by the MpHE. The increase in CD36 expression correlated with the increased phagocytic activity of microglial cells exposed to the MpHE towards amyloid β plaques. Brain slices from 5XFAD transgenic mice exposed to microglia that was previously treated with the MpHE showed a significant reduction in both the number (*p < 0*.*001*) (Fig. 10c, d) and size (*p = 0*.*03*) (Fig. 10c, e) of amyloid β plaques that remained at the end of the experiment compared with those found in the slides exposed to non-treated microglia (Fig. 10c–e). Again, this effect was prevented by the PPAR-γ inhibitor GW9662 (Fig. 10c–e). Activating microglia with the PPAR-γ agonist also resulted in a significant reduction in the number of amyloid β plaques (*p = 0*.*03*), an effect that was prevented by the PPAR-γ inhibitor (Fig. 10d). Interestingly, pioglitazone had no effect on the size of the amyloid β plaques remaining on the tissue slide (Fig. 10e). To further corroborate these data, 5XFAD transgenic mice were treated with MpHE in the absence or presence of GW9662 and the amyloid β plaques phagocytized by microglia was evaluated by flow cytometry using methoxy-X04 (Fig. 11a). MpHE in vivo administration increased the CD11b+/CD36+ population (Fig. 11b, left and middle panel) as well as the CD36 expression levels (Fig. 11b right panel) via a PPAR-γ-dependent mechanism since GW9662 prevented this induction (Fig. 11b middle and right panels). The increase in CD36 expression correlated with increased phagocytic activity of microglial cells towards amyloid β plaques in the MpHE-treated 5XFAD since we observed an increase in both the CD36^+^/methoxy-X04^+^ cells (Fig. 11c middle panel) and in the methoxy-X04 staining (Fig. 11c right panel). Importantly, the increased microglia phagocytic activity was prevented by the PPAR-γ inhibitor GW9662 (Fig. 11c, middle and right panels). These results show that MpHE enhances microglia phagocytic activity towards amyloid β plaques by promoting CD36 expression via PPAR-γ activity in the 5XFAD transgenic mice. Accordingly, MpHE in vivo administration increased PPAR-γ levels in the 5XFAD mice frontal cortex (Wt HFD vehicle *vs* Wt HFD Mp, *p* = 0.0050; 5XFAD ND vehicle *vs* 5XFAD ND Mp, *p* = 0.0934; 5XFAD HFD vehicle *vs* 5XFAD HFD Mp, *p* = 0.0036) (Fig. 12a).

As described above, MpHE contains oleanolic acid, a natural agonist of PPAR-γ (43, 44). Therefore, we tested whether the improved microglial phagocytic activity resulting from MpHE treatment involved oleanolic acid-mediated PPAR-γ activation. In accordance with this idea, oleanolic acid increased PPAR-γ levels specifically in microglia (Ctrl *vs* 4.5 μg oleanolic acid, *p* = 0.0414; Ctrl *vs* 45 μg oleanolic acid, *p* = 0.0608). Congruently with the fact that PPAR-γ induces its own expression through a positive feedback loop [45], the PPAR-γ specific inhibitor GW9662 (100 μM) prevented the increase in PPAR-γ levels resulting from the oleanolic acid treatment (4.5 μg/mL oleanolic acid *vs* GW9662 + 4.5 μg/ mL oleanolic acid (*p* = 0.0116); 45 μg/mL oleanolic acid *vs* GW9662 + 45 μg/mL oleanolic acid (*p* = 0.0079)) (Fig. 12b). Together, these results indicate that the MpHE promotes microglia phagocytosis and degradation of amyloid β plaques by inducing PPAR-γ mediated CD36 expression (Fig. 12c).

## Discussion

Plant extracts have been used as an alternative therapy to revert the AD marks. It is thought that the mixture of compounds with synergistic activities as anti-oxidant and anti-inflammatory activities favors Aβ reduction, acetylcholinesterase inhibition, and monoamines modification [48, 49]. Previously, the *M*. *parviflora* extract has been shown to present hypoglycemic, anti-oxidant, and anti-inflammatory activities [20, 21, 50] and even it has been reported to have a beneficial effect against Aβ-injected mice [51]. In the present study, we demonstrate the neuroprotective effect of a long-term treatment with MpHE in the 5XFAD mouse model of AD. This model has been widely used to examine the therapeutic value of candidate agents for AD treatment [52–54], displaying age-dependent neurological and motor deficits that mimic common phenotypes seen in AD patients, without the presence of neurofibrillary tangles [55]. In addition, we also provide evidence indicating that the MpHE attenuates the negative effect of chronic peripheral inflammation on the development of AD pathological hallmarks. The MpHE reduced adipose tissue inflammation and insulin resistance in obese mice fed with a HFD; a well-established experimental approach to induce insulin resistance [56]. Together, our results show for the first time that a long-term treatment with a MpHE attenuates both peripheral and central inflammation resulting in improved memory and learning abilities in a familial Alzheimer’s disease model. The molecular mechanism activated by the MpHE involves the activation of the PPAR-γ pathway and increased scavenger receptor CD36 expression which improves microglia phagocytic activity thus reducing Aβ load and inflammation in the CNS. Therefore, our data uncover the signaling pathway regulated by the MpHE resulting in neuroprotection.

Our phytochemical analysis identified two compounds in the MpHE, oleanolic acid and scopoletin. Interestingly, both compounds have hypoglycemic, anti-inflammatory, and anti-oxidant properties. A proposed anti-inflammatory mechanism for oleanolic acid involves the upregulation of the transcription factor Nrf2, which inhibits the expression of NF-κB [42]. As oleanolic acid, the scopoletin anti-inflammatory effect also involves the inhibition of the NF-κB and p38 MAPK pathways [57]. Congruent with oleanolic acid and scopoletin anti-NF-κB activity, here, we demonstrate that the MpHE decreases the NF-κB activation in a concentration-dependent manner. Furthermore, our ex vivo experiments show that this treatment effectively prevents microglia activation by inhibiting the morphological changes typically induced by LPS, thus confirming the anti-inflammatory effects of MpHE previously described using ear edema and vascular permeability models [21]. Additionally, it has been shown that synthetic triterpenoid analogs of oleanolic acid attenuate microglia activation and the pro-inflammatory phenotype [58].

In contrast with previous studies showing that a HFD had no effect on the Aβ plaque burden in the hippocampus from old 3xTgAD mice [59], we found that HFD increases the deposition of hippocampal β-amyloid plaques in the 5XFAD mice model. This correlated with the fact that these animals were glucose intolerant and showed insulin resistance, which is in agreement with experimental evidence indicating that the energy metabolism in the brain plays a key role in AD development [60–62]. This is consistent with previous reports that confirm impairment in hippocampal synaptic plasticity in animals fed with HFD [63–65].

Recent studies showed that a short-term HFD feeding (16 weeks) to 5XFAD mice does not induce metabolic disorder, including blood glucose levels, and does not increase body weight, although the HFD had a notorious impact on the cognition, thus considering these effects as independent phenomena [66]. However, insulin released by the pancreas crosses the blood-brain barrier and binds to and activates its specific receptor in the CNS, including those expressed in the hippocampus. Accordingly, insulin enhances memory in healthy humans and rodents [67, 68]. Thus, impaired insulin receptor signaling contributes to the development of hippocampal insulin resistance (IR). Clinical and preclinical studies suggest that hippocampal IR leads to the neuroplasticity deficits observed in both type II diabetes and AD. Recently, it has been proposed that neuroinflammation may serve as a link between aging, type II diabetes, and AD by causing IR [14, 69]. Pro-inflammatory molecules (e.g., TNF, IL-6, IL-1β) are increased in type II diabetes and AD, which may compromise the hippocampal insulin receptor expression and/or signaling. For instance, TNF may cause hippocampal IR by increasing serine phosphorylation of IRS1 [70]. Although further studies are needed to establish whether IR in the CNS regulates memory by itself or in combination with peripheral IR, experimental data support that enhancement of hippocampal insulin receptor signaling could reduce or reverse the memory deficits observed in type II diabetes and AD [71]. These observations point out the importance of establishing pharmacological and/or preventive strategies to improve cognitive deficits in individuals suffering metabolic syndrome. Interestingly, here, we show that the MpHE abolished the increase in TNF levels in the cortex of lean and obese 5XFAD transgenic mice.

Our results show also that the long-term treatment with MpHE significantly reduces the body weight gain, the impairment in the glucose metabolism in transgenic and non-transgenic mice and surprisingly, reduced the Aβ deposition in transgenic mice. Furthermore, we observed that MpHE significantly decreases the escape latency in the learning and memory tests, in accordance with a previous study where an ethanolic *M*. *parviflora* extract increased the learning and memory along with reduced reactive oxygen species (ROS) levels after injection of Aβ in mice [51]. These data are consistent with oleanoic acid and scopoletin neuroprotective effects. The oleanolic acid isolated from *Aralia cordata* confers neuroprotection in rat cortical neuronal cultures by inhibiting neuronal death and the generation of ROS induced by Aβ. Equally, the ethanolic extract of *Aralia cordata* possesses antidementia activities in mice injected intracerebroventricularly with Aβ peptides [72].

Additionally, scopoletin increases presynaptic activity-dependent acetylcholine (ACh) release, enhances hippocampal long-term potentiation (LTP), and improves cognition in cholinergically impaired and in age-impaired mice. Scopoletin neuroprotective effect might result from interacting with nicotinic ACh receptors (nAChRs) enhancing NMDA-dependent LTP [73]. Activated nAChRs can promote neuronal survival and may also decrease the synthesis and deposition of Aβ peptides [74]. If the neuroprotective effects of *Malva parviflora* involves the interaction of the scopoletin present in the extract with the nAChRs is yet to be determined.

During the development and in adulthood, the microglial cells maintain the brain homeostasis perceiving their microenvironment. The healthy state of the microglial cells is altered by the presence of soluble and aggregated Aβ, by the synaptic degeneration observed in the early AD, among other signals. This causes an increase in the production of inflammatory molecules from the microglia leading to reprograming the expression of receptors involved in the AD progression [32, 75], for example, TNF decreased the expression of scavenger receptor A (SRA) and CD36 resulting in reduced Aβ uptake [33]. Here, we identified the neuroprotector effect of MpHE against the AD progression by the ability to regulate the microglia activation and its phagocytic capacity, increasing the accumulation of ramified microglia with long and thin processes around the Aβ plaques in the cortex and hippocampus. Previous work has demonstrated that microglial primary cultures from adult mice are not compromised in their ability to recognize Aβ fibrils but have deficient phagocytic capacity when compared to that of early postnatal microglial cultures [76]. Interestingly, the MpHE increases the phagocytic index in primary microglial cultures from early postnatal and even from 8- and 10-months-old 5XFAD mice. This effect correlated with a reduction in the M1 pro-inflammatory phenotype since treatment with MpHE clearly promoted a reduction in CD86 and TNF expression levels in both lean or obese 5XFAD mice. Thus, MpHE may alleviate the negative effect of the inflammatory pathways on the insulin receptor signaling and in this way, reverse the memory deficits observed in type II diabetes and AD.

TREM-2 is a molecule directly related to the phagocytic capacity of microglial cells [77] and to the reduction of the neuronal damage produced by Aβ plaques [78]. According with the fact that TREM-2 expression is upregulated in different mice models of AD in a time-dependent manner [79], here, we observed that *M*. *parviflora* treatment tended to maintain high levels of TREM-2 expression in the cortex of 5XFAD mice at 9 months of age. Recently, it has been described a novel microglia subpopulation associated with neurodegenerative diseases (DAM) that is beneficial for AD [80]. The DAM program is activated in two-step checkpoints where microglia switch from homeostatic to stage 1 DAM (TREM2 independent) and to stage 2 DAM (TREM2 dependent) at late stages of the AD (8 months). Interestingly, the TREM2-dependent stage is associated with phagocytic activity which correlates with previous observations showing that absence of TREM2 in microglia at the late stage of AD, but not at the early stages, exacerbates the disease [81]. Whether *M*. *parviflora* accelerates the DAM phenotype (TREM2 dependent) to increase the ability of microglia to digest amyloid plaques that results in neuroprotection warrants additional investigation. Also, our results point out at CD36 as the scavenger receptor mediating microglia phagocytosis of amyloid β plaques in response to MpHE. This involves PPARγ activation, probably mediated by oleanolic acid [43, 44] present in the *M*. *parviflora* HE and the subsequent induction of CD36 expression. In agreement with previous results [45], microglia exposure to pioglitazone resulted in increased PPARγ protein levels. Interestingly, *M*. *parviflora* HE had the same effect, suggesting that pioglitazone and the *M*. *parviflora* HE activate PPARγ by a similar mechanism. Although PPARγ activation, resulting from pioglitazone exposure, promoted CD36 expression to a similar extent as that resulting from the MpHE treatment, the phagocytosis and clearance activity of pioglitazone-treated microglia towards amyloid β plaques was reduced when compared with that of microglia treated with the MpHE. This suggests that the MpHE through its anti-inflammatory effects might also activate autophagy, a mechanism that is also critical for amyloid β plaques clearance and impaired by the inflammatory environment elicited by amyloid β peptides [8].

Individuals with type II diabetes have increased risk for developing AD by at least twofold [82]. However, the precise mechanism for this association is not known. Here, we showed that alterations in glucose metabolism (i.e., IR) resulting from obesity exacerbate the memory loss induced by Aβ accumulation. Treatment with the MpHE restores insulin sensitivity and glucose tolerance in obese mice, which correlated with a diminished inflammatory environment and increased cognition in the 5XFAD mice. Additionally, we found that MpHE regulates PPAR-γ activity and mediates microglial amyloid β plaques phagocytosis. Thus, by regulating the microglia phagocytic activity via a PPAR-γ-dependent mechanism and preventing the pro-inflammatory M1 phenotype, *M*. *parviflora* reduces β-amyloid plaque load and improves learning and memory in lean and obese mice. Additionally, we do not discard the possibility that MpHE through the activation of PPARγ, prevents IKK activity [44] and therefore IRS phosphorylation on serine residues, thus improving insulin signaling directly on hippocampal neurons reversing the memory deficits observed in type II diabetes and AD. Therefore, *M*. *parviflora*-derivative compounds represent an alternative to prevent cognitive impairment associated with a metabolic disorder as well as an effective prophylactic candidate for AD progression.

## Conclusions

Peripheral inflammation resulting from obesity has been shown to exacerbate learning and memory loss in humans and in animal models of AD. Here, we show that obesity further promoted Aβ plaque formation in the 5XFAD mice. However, administration of the MpHE reversed obesity-mediated deleterious effects of Aβ deposition on memory and learning. The MpHE attenuated pro-inflammatory M1 microglia activation while sustaining anti-inflammatory M2 microglia, since CD86 and TNF expression levels observed in the cortex of 5XFAD mice fed with ND and HFD were reduced in the cortex from 5XFAD mice treated with MpHE. In parallel, MpHE tended to increase the levels of Mgl1 and from TREM2 in the cortex of lean and obese 5XFAD mice. In accordance with this, MpHE reduced the astrogliosis throughout the hippocampus of HFD-fed 5XFAD mice. In addition, MpHE increases microglia phagocytic activity via a PPAR-γ/CD36-dependent mechanism. Our data indicate that by regulating microglia phagocytic activity and by preventing the microglia pro-inflammatory M1 phenotype, *M*. *parviflora* reduces β-amyloid plaque load and improves learning and memory in lean and obese mice. Our studies also demonstrate that MpHE can prevent not only HFD-induced neuroinflammation, but also systemic inflammation since it reduces the infiltration of immune cells to the adipose tissue and delays the increase in body weight and prevents the systemic insulin resistance and glucose intolerance produced by HFD in the 5XFAD mice. Thus, by regulating the microglia phagocytic activity via a PPAR-γ/CD36-dependent mechanism and preventing the pro-inflammatory M1 phenotype, MpHE reduces the levels of pro-inflammatory molecules (e.g., TNF, IL-6), thus improving learning and memory in obese mice, which could result from restored insulin receptor signaling in hippocampal neurons. Strategies to prevent obesity-associated brain dysfunction are limited. *M*. *parviflora*-derivative compounds offer a novel adjunct therapeutic approach to ameliorate obesity-associated peripheral inflammation and neuroinflammation. Therefore, *M*. *parviflora* may be a safe and effective alternative to the conventional NSAIDs used for the treatment of neurodegenerative diseases as AD.

## Additional files

Additional file 1: **Figure S1.** *Malva parviflora* hydroalcoholic extract at three different doses protects from learning and memory deficit in LPS injected mice. (PDF 184 kb)

Additional file 2: **Figure S2.** *Malva parviflora* extract reduces adipose tissue inflammation in 5XFAD transgenic mice fed with high fat diet. (PDF 135 mb)

Additional file 3: **Figure S3.** Oleanolic acid and scopoletin inhibit LPS-induced NF-kB activity in mouse RAW-Blue macrophages. (PDF 283 kb)

## Data Availability

The data supporting the findings of this study are presented within the manuscript.

## Abbreviations

ACh: Acetylcholine
AD: Alzheimer’s disease
APP: Amyloid precursor protein
AUC: Area under the curve
Aβ: Amyloid β
DMEM: Dulbecco’s Modified Eagle Medium
GFAP: Glial fibrillary acidic protein
GTT: Glucose tolerance test
HFD: High-fat diet
IL-1β: Interleukin 1 beta
IL-6: Interleukin 6
IR: Insulin resistance
IRT: Insulin resistance test
LPS: Lipopolysaccharide
LTP: Long-term potentiation
*M*. *parviflora*: *Malva parviflora*
MpHE: *M*. *parviflora* leaf hydroalcoholic extract
nAChRs: Nicotinic ACh receptors
ND: Normal diet
NSAIDs: Non-steroidal anti-inflammatory
PBS: Phosphate buffer saline
PFA: Paraformaldehyde
PPAR-γ: Peroxisome proliferator-activated receptor gamma
ROS: Reactive oxygen species
RT: Room temperature
SEAP: Embryonic alkaline phosphatase
SRA: Scavenger receptor A
TNF: Tumor necrosis factor

## References

[1] Wortmann M (2012). Dementia: a global health priority - highlights from an ADI and World Health Organization report. Alzheimers Res Ther.

[2] Reitz C, Mayeux R (2014). Alzheimer disease: epidemiology, diagnostic criteria, risk factors and biomarkers. Biochemical Pharmacol.

[3] Latta CH, Brothers HM, Wilcock DM (2015). Neuroinflammation in Alzheimer’s disease; a source of heterogeneity and target for personalized therapy. Neuroscience.

[4] Haass C (2004). Take five--BACE and the gamma-secretase quartet conduct Alzheimer’s amyloid beta-peptide generation. EMBO J.

[5] Busciglio J, Lorenzo A, Yeh J, Yankner BA (1995). Beta-amyloid fibrils induce tau phosphorylation and loss of microtubule binding. Neuron.

[6] McGeer EG, McGeer PL (2003). Inflammatory processes in Alzheimer’s disease. Prog Neuropsychopharmacol Biol Psychiatry.

[7] Akiyama H (2000). Cell mediators of inflammation in the Alzheimer disease brain. Alzheimer Dis Assoc Disord.

[8] Alvarez-Arellano L (2018). Autophagy impairment by caspase-1-dependent inflammation mediates memory loss in response to beta-amyloid peptide accumulation. J Neurosci Res.

[9] Heneka MT (2013). NLRP3 is activated in Alzheimer’s disease and contributes to pathology in APP/PS1 mice. Nature.

[10] Farooqui AA, Farooqui T, Panza F, Frisardi V (2012). Metabolic syndrome as a risk factor for neurological disorders. Cell Mol Life Sci.

[11] Mayeux R., Stern Y. (2012). Epidemiology of Alzheimer Disease. Cold Spring Harbor Perspectives in Medicine.

[12] Biessels GJ, Staekenborg S, Brunner E, Brayne C, Scheltens P (2006). Risk of dementia in diabetes mellitus: a systematic review. Lancet Neurol.

[13] Farris W (2003). Insulin-degrading enzyme regulates the levels of insulin, amyloid beta-protein, and the beta-amyloid precursor protein intracellular domain in vivo. Proc Natl Acad Sci U S A.

[14] De Felice FG, Ferreira ST (2014). Inflammation, defective insulin signaling, and mitochondrial dysfunction as common molecular denominators connecting type 2 diabetes to Alzheimer disease. Diabetes.

[15] Song J, Lee JE (2013). Adiponectin as a new paradigm for approaching Alzheimer’s disease. Anat Cell Biol.

[16] Yamanaka M (2012). PPARgamma/RXRalpha-induced and CD36-mediated microglial amyloid-beta phagocytosis results in cognitive improvement in amyloid precursor protein/presenilin 1 mice. J Neurosci.

[17] Lim GP (2001). Ibuprofen effects on Alzheimer pathology and open field activity in APPsw transgenic mice. Neurobiol Aging.

[18] Pedraza-Alva Gustavo, Ramírez-Serrano Cristina E., Pedraza Fernando, Flores-Vallejo Rosario del Carmen, Villarreal María Luisa, Pérez-Martínez Leonor (2019). From traditional remedies to cutting-edge medicine: Using ancient mesoamerican knowledge to address complex disorders relevant to psychoneuroimmunology. Brain, Behavior, and Immunity.

[19] Pedraza-Alva G (2015). Negative regulation of the inflammasome: keeping inflammation under control. Immunol Rev.

[20] Perez Gutierrez RM (2012). Evaluation of hypoglycemic activity of the leaves of *Malva parviflora* in streptozotocin-induced diabetic rats. Food Func.

[21] Bouriche H, Meziti H, Senator A, Arnhold J (2011). Anti-inflammatory, free radical-scavenging, and metal-chelating activities of *Malva parviflora*. Pharm Biol.

[22] Tai MM (1994). A mathematical model for the determination of total area under glucose tolerance and other metabolic curves. Diabet Care.

[23] Bolte S, Cordelieres FP (2006). A guided tour into subcellular colocalization analysis in light microscopy. J Microsc.

[24] Tejerizo GT (2015). Development of molecular tools to monitor conjugative transfer in rhizobia. J Microbiol Methods.

[25] Luo Y, Isaac BM, Casadevall A, Cox D (2009). Phagocytosis inhibits F-actin-enriched membrane protrusions stimulated by fractalkine (CX3CL1) and colony-stimulating factor 1. Infect Immun.

[26] Lucin KM (2013). Microglial beclin 1 regulates retromer trafficking and phagocytosis and is impaired in Alzheimer’s disease. Neuron.

[27] Perez-Martinez L (1998). Dexamethasone rapidly regulates TRH mRNA levels in hypothalamic cell cultures: interaction with the cAMP pathway. Neuroendocrinology.

[28] Livak KJ, Schmittgen TD (2001). Analysis of relative gene expression data using real-time quantitative PCR and the 2(-Delta Delta C(T)) Method. Methods.

[29] Dineley KT, Jahrling JB, Denner L (2014). Insulin resistance in Alzheimer’s disease. Neurobiol Dis.

[30] Heneka MT (2015). Neuroinflammation in Alzheimer’s disease. Lancet Neurol.

[31] Julien C (2010). High-fat diet aggravates amyloid-beta and tau pathologies in the 3xTg-AD mouse model. Neurobiol Aging.

[32] Mhatre SD, Tsai CA, Rubin AJ, James ML, Andreasson KI (2015). Microglial malfunction: the third rail in the development of Alzheimer’s disease. Trends Neurosci.

[33] Hickman SE, Allison EK, El Khoury J (2008). Microglial dysfunction and defective beta-amyloid clearance pathways in aging Alzheimer’s disease mice. J Neurosci.

[34] Malm TM, Jay TR, Landreth GE (2015). The evolving biology of microglia in Alzheimer’s disease. Neurotherapeutics.

[35] Bolmont T (2008). Dynamics of the microglial/amyloid interaction indicate a role in plaque maintenance. J Neuroscience.

[36] Stewart CR (2010). CD36 ligands promote sterile inflammation through assembly of a Toll-like receptor 4 and 6 heterodimer. Nat Immunol.

[37] Tam WY, Ma CH (2014). Bipolar/rod-shaped microglia are proliferating microglia with distinct M1/M2 phenotypes. Sci Rep.

[38] Walker DG, Lue LF (2015). Immune phenotypes of microglia in human neurodegenerative disease: challenges to detecting microglial polarization in human brains. Alzheimers Res Ther.

[39] McCarthy RC (2016). Characterization of a novel adult murine immortalized microglial cell line and its activation by amyloid-beta. J Neuroinflammation.

[40] Takahashi K, Rochford CD, Neumann H (2005). Clearance of apoptotic neurons without inflammation by microglial triggering receptor expressed on myeloid cells-2. J Exp Med.

[41] Chang WC (2015). Scopoletin protects against methylglyoxal-induced hyperglycemia and insulin resistance mediated by suppression of advanced glycation endproducts (AGEs) generation and anti-glycation. Molecules.

[42] Camer D, Yu Y, Szabo A, Huang XF (2014). The molecular mechanisms underpinning the therapeutic properties of oleanolic acid, its isomer and derivatives for type 2 diabetes and associated complications. Mol Nutr Food Res.

[43] Kang X (2017). Oleanolic acid prevents cartilage degeneration in diabetic mice via PPARgamma associated mitochondrial stabilization. Biochem Biophys Res Commun.

[44] Croasdell A (2015). PPARgamma and the innate immune system mediate the resolution of inflammation. PPAR Res.

[45] Pelzer T (2005). Pioglitazone reverses down-regulation of cardiac PPARγ expression in Zucker diabetic fatty rats. Biochem Biophis Res Commun.

[46] Leesnitzer LM (2002). Functional consequences of cysteine modification in the ligand binding sites of peroxisome proliferator activated receptors by GW9662. Biochemistry.

[47] Tontonoz P, Nagy L, Alvarez JG, Thomazy VA, Evans RM (1998). PPARgamma promotes monocyte/macrophage differentiation and uptake of oxidized LDL. Cell.

[48] Obulesu M, Rao DM (2011). Effect of plant extracts on Alzheimer’s disease: an insight into therapeutic avenues. J Neurosci Rural Pract.

[49] Kure C, Timmer J, Stough C (2017). The immunomodulatory effects of plant extracts and plant secondary metabolites on chronic neuroinflammation and cognitive aging: a mechanistic and empirical review. Front Pharmacol.

[50] Farhan H, Rammal H, Hijazi A, Hamad H, Daher A, Reda M, Badran B (2012). In vitro antioxidant activity of ethanolic and aqueous extracts from crude *Malva parviflora* L. grown in Lebanon. Asian J Pharmaceutic Clin Res.

[51] Aslam M, Sial AA (2014). Neuroprotective effect of ethanol extract of leaves of *Malva parviflora* against amyloid-beta- (Abeta-) mediated Alzheimer’s disease. Int Sch Res Notices.

[52] Zeng Y (2015). Tripchlorolide improves cognitive deficits by reducing amyloid beta and upregulating synapse-related proteins in a transgenic model of Alzheimer’s Disease. J Neurochem.

[53] Park H (2014). Anti-amyloidogenic effects of ID1201, the ethanolic extract of the fruits of *Melia toosendan*, through activation of the phosphatidylinositol 3-kinase/Akt pathway. Environ Toxicol Pharmacol.

[54] Cho WH (2014). ID1201, the ethanolic extract of the fruit of *Melia toosendan* ameliorates impairments in spatial learning and reduces levels of amyloid beta in 5XFAD mice. Neurosci Lett.

[55] Oakley H (2006). Intraneuronal beta-amyloid aggregates, neurodegeneration, and neuron loss in transgenic mice with five familial Alzheimer’s disease mutations: potential factors in amyloid plaque formation. J Neurosci.

[56] Ho L (2004). Diet-induced insulin resistance promotes amyloidosis in a transgenic mouse model of Alzheimer’s disease. FASEB J.

[57] Pereira Dos Santos Nascimento MV (2016). Inhibition of the NF-kappaB and p38 MAPK pathways by scopoletin reduce the inflammation caused by carrageenan in the mouse model of pleurisy. Immunopharmacol Immunotoxicol.

[58] Tran TA, McCoy MK, Sporn MB, Tansey MG (2008). The synthetic triterpenoid CDDO-methyl ester modulates microglial activities, inhibits TNF production, and provides dopaminergic neuroprotection. J Neuroinflammation.

[59] Knight EM, Martins IV, Gumusgoz S, Allan SM, Lawrence CB (2014). High-fat diet-induced memory impairment in triple-transgenic Alzheimer’s disease (3xTgAD) mice is independent of changes in amyloid and tau pathology. Neurobiol Aging.

[60] Moser VA, Pike CJ (2017). Obesity accelerates alzheimer-related pathology in APOE4 but not APOE3 mice. eNeuro.

[61] Petrov D (2015). High-fat diet-induced deregulation of hippocampal insulin signaling and mitochondrial homeostasis deficiences contribute to Alzheimer disease pathology in rodents. Biochim Biophys Acta.

[62] Vandal M (2014). Insulin reverses the high-fat diet-induced increase in brain Abeta and improves memory in an animal model of Alzheimer disease. Diabetes.

[63] Liu Z (2015). High-fat diet induces hepatic insulin resistance and impairment of synaptic plasticity. PloS One.

[64] Spinelli M (2017). Brain insulin resistance impairs hippocampal synaptic plasticity and memory by increasing GluA1 palmitoylation through FoxO3a. Nat Commun.

[65] Stranahan AM (2008). Diet-induced insulin resistance impairs hippocampal synaptic plasticity and cognition in middle-aged rats. Hippocampus.

[66] Lin B, et al. High-fat-diet intake enhances cerebral amyloid angiopathy and cognitive impairment in a mouse model of Alzheimer’s disease, independently of metabolic disorders. J Am Heart Assoc. 2016;5(6). 10.1161/JAHA.115.003154 PMC4937262 27412896

[67] Stern SA, Chen DY, Alberini CM (2014). The effect of insulin and insulin-like growth factors on hippocampus- and amygdala-dependent long-term memory formation. Learn Mem.

[68] Moosavi M, Naghdi N, Choopani S (2007). Intra CA1 insulin microinjection improves memory consolidation and retrieval. Peptides.

[69] Tucsek Z (2014). Obesity in aging exacerbates blood-brain barrier disruption, neuroinflammation, and oxidative stress in the mouse hippocampus: effects on expression of genes involved in beta-amyloid generation and Alzheimer’s disease. J Gerontol A Biol Sci Med Sci.

[70] Bomfim TR (2012). An anti-diabetes agent protects the mouse brain from defective insulin signaling caused by Alzheimer’s disease- associated Abeta oligomers. J Clin Invest.

[71] Biessels GJ, Reagan LP (2015). Hippocampal insulin resistance and cognitive dysfunction. Nat Rev Neurosci.

[72] Cho SO (2009). *Aralia cordata* protects against amyloid beta protein (25-35)-induced neurotoxicity in cultured neurons and has antidementia activities in mice. J Pharmacol Sci.

[73] Hornick A (2011). The coumarin scopoletin potentiates acetylcholine release from synaptosomes, amplifies hippocampal long-term potentiation and ameliorates anticholinergic- and age-impaired memory. Neuroscience.

[74] Buckingham SD, Jones AK, Brown LA, Sattelle DB (2009). Nicotinic acetylcholine receptor signalling: roles in Alzheimer’s disease and amyloid neuroprotection. Pharmacol Rev.

[75] Heneka MT, O’Banion MK (2007). Inflammatory processes in Alzheimer’s disease. J Neuroimmunol.

[76] Njie EG (2012). Ex vivo cultures of microglia from young and aged rodent brain reveal age-related changes in microglial function. Neurobiol Aging.

[77] Kleinberger G (2014). TREM2 mutations implicated in neurodegeneration impair cell surface transport and phagocytosis. Sci Transl Med.

[78] Wang Y (2016). TREM2-mediated early microglial response limits diffusion and toxicity of amyloid plaques. J Exp Med.

[79] Brendel M (2017). Increase of TREM2 during Aging of an Alzheimer’s disease mouse model is paralleled by microglial activation and amyloidosis. Front Aging Neurosci.

[80] Keren-Shaul H (2017). A unique microglia type associated with restricting development of Alzheimer’s disease. Cell.

[81] Wang Y (2015). TREM2 lipid sensing sustains the microglial response in an Alzheimer’s disease model. Cell.

[82] Sims-Robinson C, Kim B, Rosko A, Feldman EL (2010). How does diabetes accelerate Alzheimer disease pathology?. Nat Rev Neurol.

