# Supplementary material for: Malva parviflora extract ameliorates the deleterious effects of a high fat diet on the cognitive deficit in a mouse model of Alzheimer’s disease by restoring microglial function via a PPAR-γ-dependent mechanism
Source: J Neuroinflammation. 2019 Jul 10;16:143. doi: 10.1186/s12974-019-1515-3 (PMC6617588; doi:10.1186/s12974-019-1515-3)
Supplement: Supplementary file 1 — Figure S1. Malva parviflora hydroalcoholic extract at three different doses protects from learning and memory deficit in LPS injected mice. (PDF 184 kb) [file 12974_2019_1515_MOESM1_ESM.pdf]

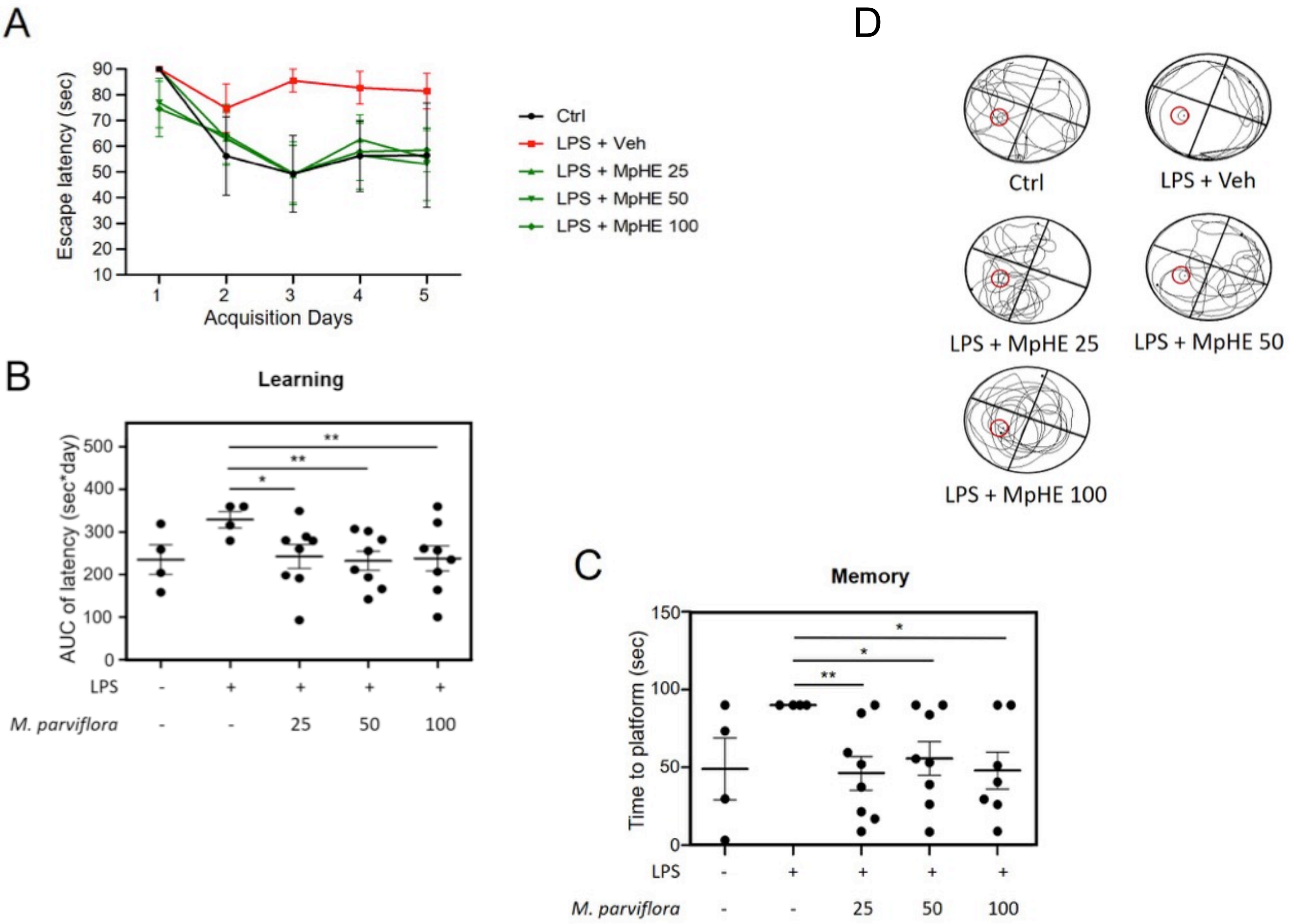

Additional file 1: Figure S1. ***Malva parviflora* hydroalcoholic extract at three different doses protects from learning and memory deficit in LPS injected mice.**

Morris Water Maze test was performed to evaluate spatial learning and memory in mice treated with different doses of *Malva parviflora* hydroalcoholic extract **(**MpHE). CD1 male mice were intraperitoneally injected with 125 µg/kg of LPS or vehicle as control for seven consecutive days. LPS injected mice then received 25, 50 or 100 mg/kg/day of the MpHE or water (vehicle) intragastrically during seven days. **(A**) Time (sec) need to reach the hidden platform (escape latency) during the five acquisition days (test trial) of control mice group (Ctrl) (●) and LPS injected mice treated with different doses of MpHE: 25 mg/kg/day (LPS + MpHE 25) (▲), 50 mg/kg/day (LPS + MpHE MpHE 50) (▼) or 100 mg/kg/day (LPS + MpHE 100) (⧫); or just vehicle (LPS + Veh) (■). Data are shown as mean ± SEM, n=4-8 animals per group. Statistical analysis was performed by two-way ANOVA with repeated measures followed by post hoc Tukey's multiple comparisons test. (**B)** Area under the curve (AUC) of the latencies for each group was calculated using the trapezoidal rule. Data are shown as mean ± SEM, n=4-8 animals per group. Statistical analysis was performed by unpaired parametric t-test with Welch correction in Prism 8. This analysis revealed a significant difference between LPS + Veh group and LPS + MpHE 25 $p=0.0321$, LPS + MpHE 50 $p=0.0100$ and LPS + MpHE 100 $p=0.0302$ groups. **(C)** Time (sec) to platform for each group during the probe trial (day eight) in the absence of platform. Statistical analysis was performed by unpaired parametric t-test with Welch correction in Prism 8. This analysis revealed a significant difference between LPS + Veh group and LPS + MpHE 25 $p=0.005$, LPS + MpHE 50 $p=0.0163$ and LPS + MpHE 100 $p=0.0128$ groups. **(D)** Representative swimming paths of mice during the probe trial on day eight are depicted. The hidden platform was located on the NW quadrant.
