# Supplementary material for: Malva parviflora extract ameliorates the deleterious effects of a high fat diet on the cognitive deficit in a mouse model of Alzheimer’s disease by restoring microglial function via a PPAR-γ-dependent mechanism
Source: J Neuroinflammation. 2019 Jul 10;16:143. doi: 10.1186/s12974-019-1515-3 (PMC6617588; doi:10.1186/s12974-019-1515-3)
Supplement: Supplementary file 2 — Figure S2. Malva parviflora extract reduces adipose tissue inflammation in 5XFAD transgenic mice fed with high fat diet. (PDF 135 mb) [file 12974_2019_1515_MOESM2_ESM.pdf]

A

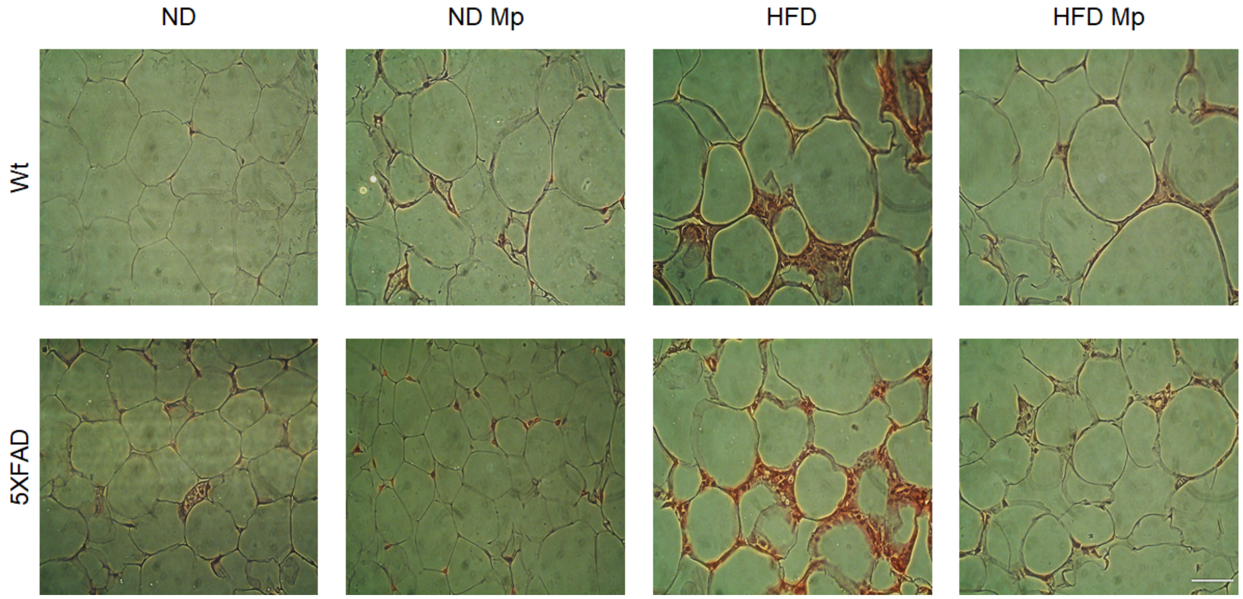


B

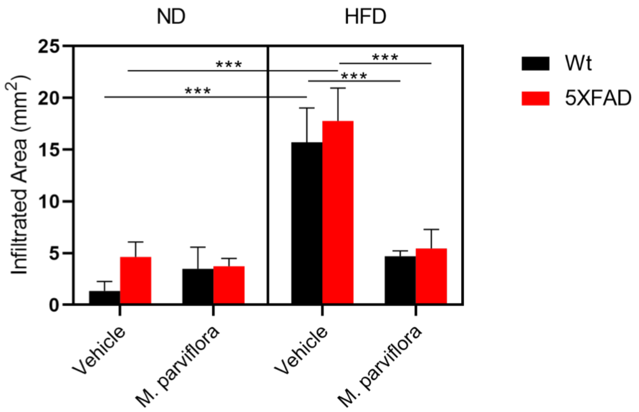


Additional file 2: Figure S2. ***Malva parviflora*** **extract reduces adipose tissue inflammation in 5XFAD transgenic mice fed with high fat diet.** Wt or 5XFAD mice were fed with either normal diet (ND) or high fat diet (HFD) and received intragastrically water (vehicle) or 50 mg/kg/day of MpHE (Mp) for eight months. Mice were sacrificed and peritoneal adipose tissue was collected, snap frozen or fixed in paraformaldehyde. **(A)** The cellular infiltration was evaluated in adipose tissue sections stained with hematoxylin and eosin under light microscopy. **(B)** The infiltrated area was determined using ImageJ software as described under materials and methods. Data are shown as mean ± SEM, n=3 animals per group. Statistical analysis was performed by three-way ANOVA followed by Tukey's multiple comparisons test. This analysis revealed a significant effect for the *M. parviflora* treatment $F(1,16)=45.09$, $p<0.0001$; for the diet $F(1,16)=85.59$, $p<0.0001$; for the interaction between *M. parviflora* treatment and diet $F(1,16)=55.94$, $p<0.0001$.
