# Supplementary material for: Malva parviflora extract ameliorates the deleterious effects of a high fat diet on the cognitive deficit in a mouse model of Alzheimer’s disease by restoring microglial function via a PPAR-γ-dependent mechanism
Source: J Neuroinflammation. 2019 Jul 10;16:143. doi: 10.1186/s12974-019-1515-3 (PMC6617588; doi:10.1186/s12974-019-1515-3)
Supplement: Supplementary file 3 — Figure S3. Oleanolic acid and scopoletin inhibit LPS-induced NF-kB activity in mouse RAW-Blue macrophages. (PDF 283 kb) [file 12974_2019_1515_MOESM3_ESM.pdf]

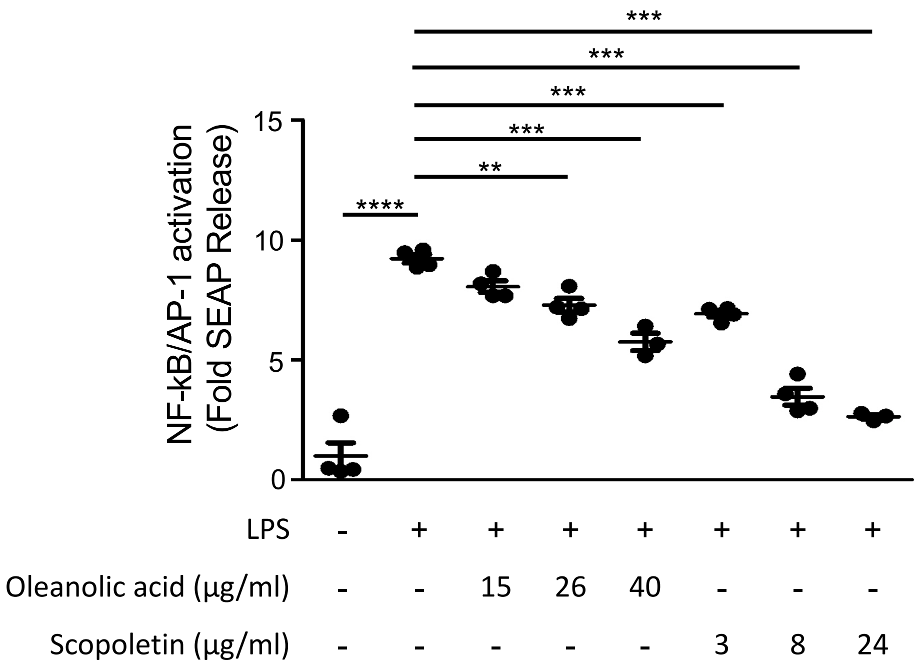

Additional file 3: Figure S3. **Oleanolic acid and scopoletin inhibit LPS-induced NF-κB activity in mouse RAW-Blue macrophages.** RAW-Blue macrophages were untreated or treated with lipopolysaccharide (LPS; 100 ng/mL) alone or together with three different concentrations of oleanolic acid (15, 26, 40 µg/mL), scopoletin (3, 8, 24 µg/mL) or the used vehicle to dissolve them (DMSO < 0.2% or Tween 20 < 0.03%, respectively). Twelve hours later, embryonic alkaline phosphatase (SEAP) activity (driven by NF-κB/AP-1 activation) was determined in the supernatants as described in materials and methods. Values are expressed as fold increase relative to SEAP reporter activity in untreated control cells. Data are shown as mean ± SEM. Statistical analysis was performed by one-way ANOVA with repeated measures followed by post hoc Tukey's multiple comparisons test. This analysis revealed a significant effect for oleanolic acid treatment at the 20 $p<0.01$ and 40 µg/mL $p<0.001$ concentrations, and for scopoletin, at the 3 $p<0.001$, 8 $p<0.001$ and 24 µg/mL $p<0.001$ concentrations.
